# An NK Cell Perforin Response Elicited via IL-18 Controls Mucosal Inflammation Kinetics during *Salmonella* Gut Infection

**DOI:** 10.1371/journal.ppat.1005723

**Published:** 2016-06-24

**Authors:** Anna A. Müller, Tamas Dolowschiak, Mikael E. Sellin, Boas Felmy, Carolin Verbree, Sandra Gadient, Alexander J. Westermann, Jörg Vogel, Salome LeibundGut-Landmann, Wolf-Dietrich Hardt

**Affiliations:** 1 Institute of Microbiology, ETH Zürich, Zürich, Switzerland; 2 Department of Cell and Molecular Biology, Microbiology, Uppsala University, Uppsala, Sweden; 3 Institute for Molecular Infection Biology, University of Würzburg, Würzburg, Germany; 4 Institute of Virology, University of Zürich, Zürich, Switzerland; University of California Davis School of Medicine, UNITED STATES

## Abstract

*Salmonella* Typhimurium (*S*.Tm) is a common cause of self-limiting diarrhea. The mucosal inflammation is thought to arise from a standoff between the pathogen's virulence factors and the host's mucosal innate immune defenses, particularly the mucosal NAIP/NLRC4 inflammasome. However, it had remained unclear how this switches the gut from homeostasis to inflammation. This was studied using the streptomycin mouse model. *S*.Tm infections in knockout mice, cytokine inhibition and –injection experiments revealed that caspase-1 (not -11) dependent IL-18 is pivotal for inducing acute inflammation. IL-18 boosted NK cell chemoattractants and enhanced the NK cells' migratory capacity, thus promoting mucosal accumulation of mature, activated NK cells. NK cell depletion and *Prf* ^-/-^ ablation (but not granulocyte-depletion or T-cell deficiency) delayed tissue inflammation. Our data suggest an NK cell perforin response as one limiting factor in mounting gut mucosal inflammation. Thus, IL-18-elicited NK cell perforin responses seem to be critical for coordinating mucosal inflammation during early infection, when *S*.Tm strongly relies on virulence factors detectable by the inflammasome. This may have broad relevance for mucosal defense against microbial pathogens.

## Author Summary

*Salmonella* Typhimurium is a common cause of foodborne diarrhea. The disease symptoms arise already a few hours after infection. However, it had remained unclear how the immune system can mount the responses eliciting the disease symptoms so quickly. Earlier work in a mouse model had shown that the gut epithelium expresses a sensor, called NAIP/NLRC4/caspase-1 inflammasome that can detect the pathogen and mount a defense by 12-18h p.i. However, it has remained uncharacterized how inflammasome sensing drives the initial gut inflammation. Here, we found that the caspase-1 inflammasome triggers the production of IL-18, a pro-inflammatory cytokine that appears essential for the early onset of inflammation. IL-18 is driving the accumulation of NK cells into the infected mucosa, via the upregulation of NK cell chemoattractants and by the stimulation of their migratory capacity. Mature NK cells seem to induce mucosal inflammation via a perforin-mediated cytotoxic response. These data suggest that the inflammasome/IL-18/NK cell axis is a driver of early mucosal inflammation via a perforin-dependent cytotoxic NK cell response. Future work will have to address, if this mechanism is equally potent in the human gut and may contribute to ramping up the host's response during the first hours of infection. This may have implications for other gut infections and might provide leads for developing therapies.

## Introduction

The intestinal mucosa is a key site limiting microbial access to the body [1, 2]. Nonetheless, some enteropathogenic bacteria, including *Salmonella enterica* subspecies 1 serovar Typhimurium (*S*.Tm), have the capacity to overcome the mucosal defenses and utilize the gut as a port of entry. It is still not well understood how defenses are mounted and coordinated to limit infection.

The innate immune system provides formidable protection against the vast majority of invading microbes. Its chemo-sensors, termed danger recognition receptors, are detecting conserved microbial products (including key virulence factors) and tissue damage inflicted by the infection, boost antimicrobial defense and recruit phagocytic cell populations to eliminate the pathogen and cellular debris [3–5]. It appears that some "successful" pathogens have evolved mechanisms to evade these innate responses [6, 7]. This is thought to complicate the analysis of many important recognition events. For example, *S*.Tm can downregulate expression of flagella and the SPI-1 type III secretion system at systemic sites and may thereby evade (at least partially) the detection by danger recognition receptors, e.g. the NAIP/NLRC4 inflammasome [8–10]. Such "stealthy" behavior is not an option at sites where the respective virulence factors (or other recognized components) are needed by the pathogen for performing an important step of the infection cycle. Based on these considerations, enteropathogenic bacteria may be particularly prone to detection by the innate immune system when they arrive at the mucosal surface and have to deploy virulence factors to achieve host cell manipulation and invasion. Indeed, recent work has identified the epithelial NAIP/NLRC4 inflammasome as a central player in the initial defense against *Citrobacter rodentium* and *S*.Tm, initiating the expulsion of infected enterocytes into the gut lumen [11–13]. However, it had remained unclear whether and how this is coordinated with other defenses at this critical site.

*S*.Tm is a key cause of diarrhea worldwide [14]. The streptomycin mouse model for *Salmonella* Typhimurium diarrhea is used to study the pathogen's virulence factors and the mucosal responses mounted upon infection [15, 16]. In the gut lumen, *S*.Tm relies on flagella to reach the epithelial surface and the TTSS-1 to bind and invade the intestinal epithelium [12, 17–21]. In fact, enterocyte invasion was already described in pioneering work employing guinea-pig and bovine models [22, 23]. While numerous mucosal immune defense mechanisms have been proposed [16, 24], the critical responses coordinating the mucosal defenses during the initial wave of pathogen invasion have remained elusive. Only recently, first mechanistic insights have been obtained in the mouse model. This has established a critical function of the epithelial NAIP/NLRC4-inflammasome to detect enterocyte invasion by *S*.Tm during the first hours of infection [12]. This inflammasome-dependent defense appears to be two-pronged. By facilitating the expulsion of infected epithelial cells into the gut lumen, the innate defense was shown to reduce epithelial pathogen loads by as much as 100-fold. However, this fails to completely clear the pathogen from the tissue. In parallel, the inflammasome response triggers a pro-inflammatory program that leads to overt tissue inflammation characterized by crypt abscesses, tissue damage and leukocyte infiltration—typical hallmarks of acute *S*.Tm diarrhea [16, 25]. However, it has remained unclear how inflammasome activation and induction of mucosal inflammation are linked in this disease.

Here, we have analyzed the mechanisms switching the naïve mucosa into overt inflammation in response to *S*.Tm infection. Our work identifies a key role of caspase-1 (not -11) mediated IL-18 production, IL-18-mediated accumulation of mature natural killer (NK) cells in the mucosa and suggests that NK-cell perforin is important for mounting gut inflammation within 12h of the infection.

## Results

### IL-18 is necessary for early stimulation of the mucosal inflammatory response to *S*.Tm

**Fig 1:**
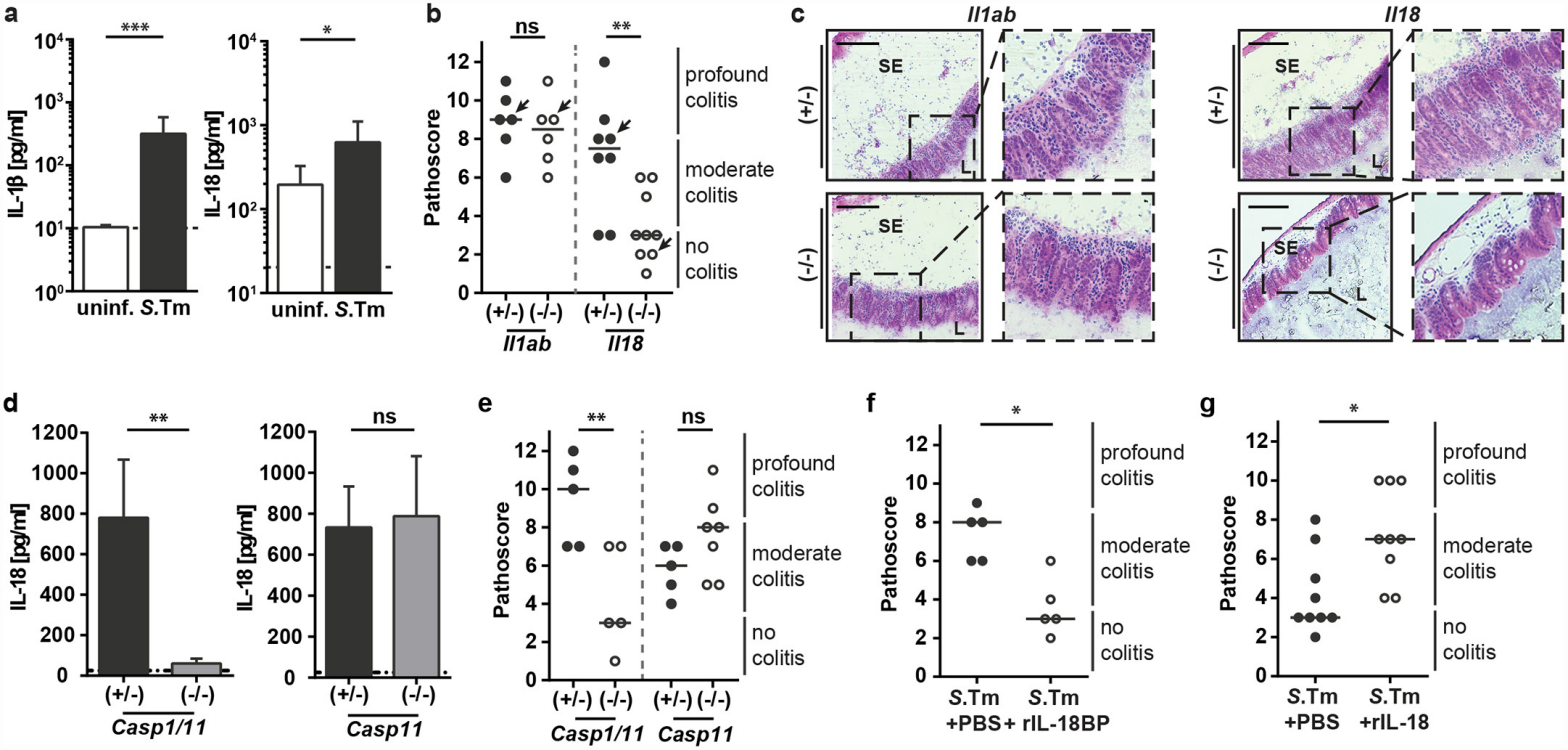
IL-18 modulates the onset of *S*.Tm-induced intestinal inflammation. (a) Mature IL-1β and IL-18 protein levels were measured in whole cecum tissue homogenates from C57BL/6 WT mice. Mice were Sm-pretreated and remained uninfected or were infected orally with 5x10^7^ CFU S.Tm for 12h (n = 7 per group); dashed lines indicate the detection limit. (b and c) *Il1ab* ^*-/-*^ and *Il18* ^*-/-*^ mice and littermate controls were Sm-pretreated and infected orally with 5x10^7^ CFU *S*.Tm for 12h (n = 6–9 per group). (b) pathological score; arrows indicate representative mice depicted in panel c, (c) HE-stained cryosections from representative mice of each group; SE = submucosal edema, L = lumen; scale bar = 100μm; left panel: *Il1ab* ^*-/-*^, right panel: *Il18* ^*-/-*^. (d and e) *Casp1/11* ^*-/-*^ and *Casp11* ^*-/-*^ mice and littermate controls were Sm-pretreated and infected orally with 5x10^7^ CFU *S*.Tm for 12h (n = 5–7 per group). (d) Mature IL-18 protein levels in whole cecum tissue lysates from *Casp1/11* ^*-/-*^ or *Casp11* ^*-/-*^ mice and littermate controls; dashed lines indicate the detection limit. (e) Pathological score. (f) C57BL/6 WT mice were Sm-pretreated, injected intraperitoneally with rIL-18BP or PBS, infected orally with 5x10^7^ CFU *S*.Tm for 12h (n = 5 per group) and mucosal inflammation was quantified. (g) C57BL/6 WT mice were Sm-pretreated, injected intraperitoneally with rIL18 or PBS, infected orally with 5x10^7^ CFU *S*.Tm for 8h (n = 9 per group) and mucosal inflammation was quantified. Data represent the mean ± SD and statistical analysis was performed using the Mann-Whitney-U test (ns = not significant, *: p<0.05; **: p<0.01; ***: p<0.001).

We set out to characterize the initiation of a mucosal immune response and the subsequent cecum inflammation upon acute *S*.Tm infection using the streptomycin mouse model [15]. As inflammasome recognition of *S*.Tm has already been identified as an important component of the early mucosal defense [12], we postulated that the inflammasome dependent IL-1 family cytokines IL-1β and IL-18 could play a role in shaping the early immune response and the onset of inflammation. Indeed, both cytokines showed elevated mature protein levels in the infected mucosal tissue already early during infection (5x10^7^ CFU *S*.Tm, 12h p.i.; Fig 1a). In case of *Il1b* this went along with a transcriptional upregulation (S1a Fig), as observed previously [26, 27]. In contrast, the IL-18 response occurred mostly at the post-transcriptional level, as *Il18* transcript levels remained unchanged at least at this early stage of the infection (S1a Fig).

To study the functional importance of IL-1β and IL-18 in the onset of mucosal inflammation, we infected mice deficient in IL-1α/β or IL-18 and appropriate littermate controls and measured the degree of mucosal inflammation using a well-established histopathological scoring scheme that assesses the degree of submucosal edema, the integrity of the epithelial layer, the abundance of mucin-filled goblet cells in the epithelial lining and the infiltration by polymorphonuclear leukocytes (according to [15]). At 12h p.i., *S*.Tm had reached equally high pathogen densities in the cecal lumen of all experimental groups (S1b Fig). IL-1α/β-deficient animals and controls featured equivalent levels of pronounced cecum pathology (Fig 1b and 1c and S1c Fig). In contrast, the cecal mucosa of the IL-18-deficient mice was much less inflamed (Fig 1b and 1c and S1c Fig). This provided a first indication that IL-18 is functionally important for initiating gut mucosal inflammation. However, the underlying molecular and cellular processes remained to be established.

Depending on the disease model, IL-18 can be processed and released via caspase-1 and/or caspase-11 inflammasomes [12, 28–30]. To identify which caspase is required for IL-18 processing in our model, we infected mice deficient in caspase-1 and -11 or deficient in caspase-11 alone and corresponding littermates and assessed mucosal IL-18 levels as well as cecal pathology. While caspase-1/11-deficient animals displayed reduced levels of mature IL-18 in the infected cecal mucosa (p<0.05; Fig 1d, left side), we observed no such reduction in the caspase-11-deficient mice (Fig 1d, right side). Correspondingly, caspase-1/11-deficient animals, but not caspase-11-deficient mice, showed reduced cecum mucosal inflammation (Fig 1e). This pheno-copied the IL-18-deficient mice (Fig 1b and 1c right side) and suggested that caspase-1 (not caspase-11) is required for IL-18 processing and the subsequent elicitation of gut inflammation during the first hours of *S*.Tm infection.

The dependency of mucosal inflammation on IL-18 was further explored in a time course experiment, infecting IL-18-deficient mice and littermates for 6h, 12h, 18h or 36h with *S*.Tm. Quantification of mature IL-18 protein in the cecum mucosa of the IL-18 proficient littermate controls revealed elevated protein levels during the first 6h-18h p.i., which returned to basal expression at 36h p.i. (S1d Fig). Importantly, colitis was clearly reduced in IL-18-deficient mice up to 18h p.i. (S1f Fig), while cecum luminal colonization appeared stable throughout the course of infection (S1e Fig). By 36h p.i., the knockout mice featured an equivalent degree of inflammation as their littermate controls (S1f Fig). Therefore, IL-18 deficiency causes a delayed onset, but not a complete blunting of mucosal inflammation in response to *S*.Tm infection.

It had remained unclear, if the observed delay in cecal pathology is explained either by an important function of IL-18 during early infection, or by a general alteration of mucosal homeostasis in the knockout mice. To address the underlying mechanism, we examined if neutralization of IL-18 during acute *S*.Tm infection would might reduce cecum pathology by 12h p.i.. To this end, we artificially increased the concentration of a naturally occurring inhibitor of IL-18, by injecting C57BL/6 wild-type mice (WT) with recombinant IL-18BP (2mg/kg i.p.) or PBS as a control. While the treatment did not affect cecum luminal colonization (S1h Fig), IL-18 inhibition significantly reduced mucosal pathology (Fig 1f). Cytokine neutralization yielded in fact an equivalent phenotype as genetic IL-18 ablation (compare Fig 1b and 1f). This is in line with a direct regulatory function of IL-18 during acute infection rather than a homeostatic imbalance in the genetically deficient animals.

Next, we examined the effect of increased IL-18 concentrations. As IL-18 accelerates the progress of inflammation (see S1f Fig), artificially raising IL-18 levels should enhance inflammation during the first hours of infection. Indeed, the injection of recombinant IL-18 (120μg/kg i.p.) enhanced mucosal inflammation by 8h p.i., an early time point when untreated mice showed only minor signs of pathology (Fig 1g). Based on this observation, we tested if IL-18 would be sufficient to drive mucosal inflammation even in the absence of invasive bacteria. To this end, we infected mice with an avirulent isogenic *S*.Tm mutant (*S*.Tm^avir^; *ΔinvG; sseD*::*aphT*; [31]), which colonizes the gut lumen but fails to invade the cecum tissue and does not elicit mucosal pathology within 4 days of infection [31, 32]. However, injection of rIL-18 during infection with *S*.Tm^avir^ did not induce mucosal inflammation (S1k Fig). From these findings, we conclude that IL-18 is required for, and modulates the potency of the acute inflammatory response towards invasive *S*.Tm. Yet, it is insufficient to induce inflammation in the absence of pathogen tissue invasion.

### Neutrophils are recruited to the infected cecal LP in an IL-18 dependent manner but do not accelerate the onset of gut inflammation

**Fig 2:**
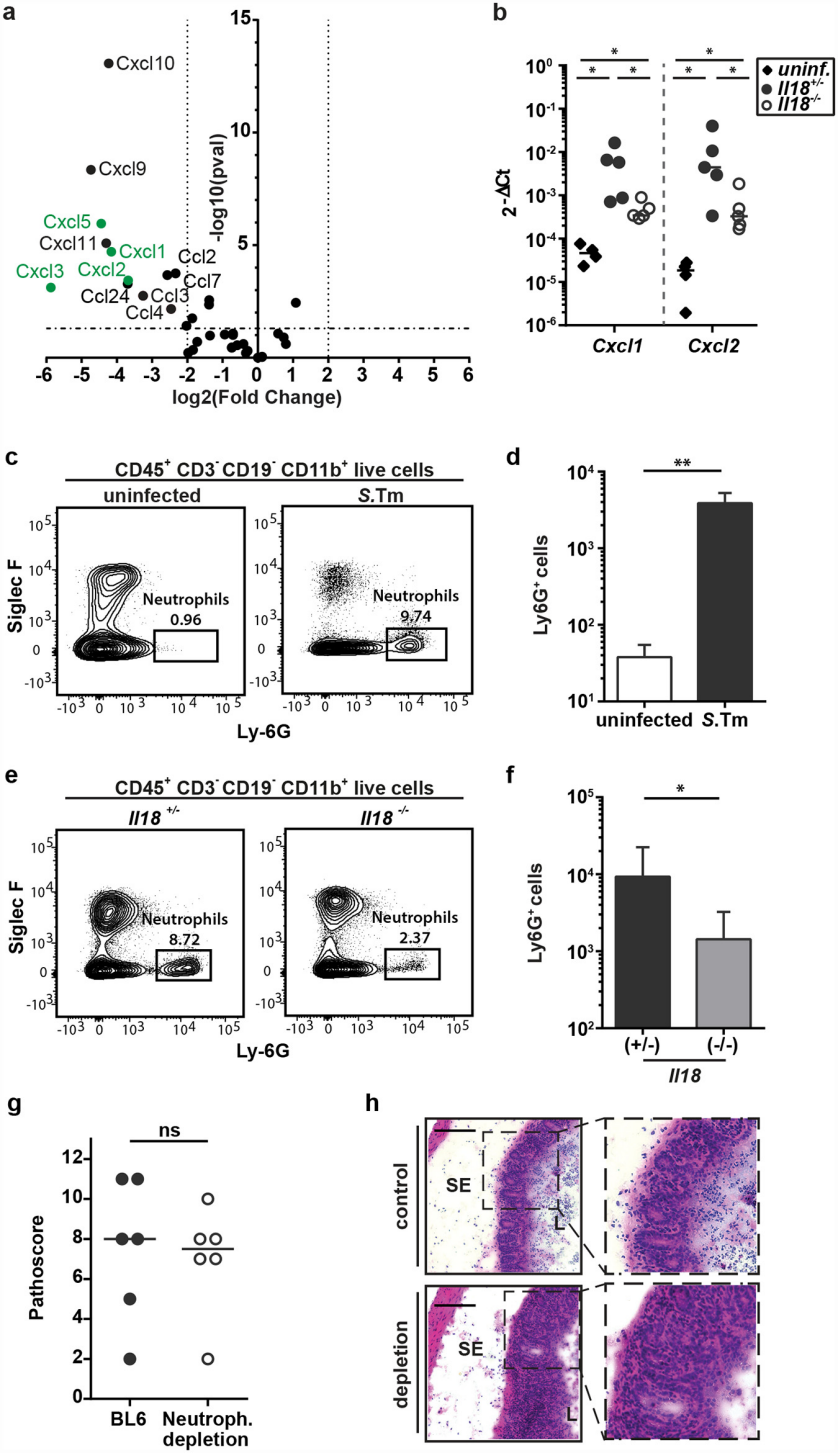
Neutrophil recruitment into the infected mucosa is decreased in absence of IL-18 but neutrophil depletion does not seem to be a main driver during the initiation of mucosal inflammation. (a) *Il18* ^*-/-*^ mice and littermate controls were Sm-pretreated, infected orally with 5x10^7^ CFU *S*.Tm for 12h (n = 3–4 per group). RNA-Seq was performed on RNA extracted from complete cecum tissue. RNA-Seq analysis: The Volcano plot shows the induction (log_2_ fold change) versus the -log_10_ p-value for all chemokines. Chemokines able to induce neutrophil recruitment are highlighted in green. (b) C57BL/6 WT mice and Il18^-/-^ mice and littermates were Sm-pretreated and either uninfected (WT) or infected orally with 5x10^7^ CFU *S*.Tm for 12h (n = 4–5 per group). *Cxcl1 and Cxcl2* mRNA levels in whole cecum tissue were measured by RT-qPCR. Results are presented relative to the expression of *Actb*. (c and d) Flow cytometric analysis of isolated cecal LP cells from Sm-pretreated C57BL/6 WT mice, either uninfected or infected with 5x10^7^ CFU *S*.Tm (n = 6 per group). Single live cells were gated on CD45^+^CD3^-^CD19^-^CD11b^+^ cells. (c) Representative dot plots and (d) quantification of Ly-6G^+^ cells. (e and f) Flow cytometric analysis of isolated cecal LP cells from *Il18* ^-/-^ mice and littermates, Sm-pretreated and orally infected with 5x10^7^ CFU *S*.Tm for 12h (n = 8 per group). Single live cells were gated on CD45^+^CD3^-^CD19^-^CD11b^+^ cells. (e) Representative dot plots and (f) quantification of Ly-6G^+^ cells. (g and h) C57BL/6 WT mice were injected intraperitoneally with anti-G-CSF (0.4mg/kg; two consecutive days) and anti-Ly-6G (6mg/kg; two days prior to infection) or PBS. Mice were Sm-pretreated and infected orally with 5x10^7^ CFU *S*.Tm for 12h (n = 6 per group). (g) Pathological score and (h) representative HE-stained cryosection. Data represent the mean ± SD and statistical analysis was performed using the Mann-Whitney-U test (ns = not significant, *: p<0.05; **: p<0.01).

Cytokine and chemokine response patterns can provide cues about underlying immunological processes. To elucidate how IL-18 affects the mounting of gut inflammation, we therefore collected infected cecum tissue samples from IL-18-deficient mice and littermate controls (12h p.i., 5x10^7^ CFU *S*.Tm) and performed transcriptional profiling by RNA-Seq. In total, mRNA levels for more than 200 genes were reduced in IL-18-deficient mice compared to the littermate controls (for a complete list of significantly regulated genes, see S1 Table). Many of those differentially expressed genes belonged to pathways involved in general pro-inflammatory responses, immunity to infection as well as chemokine and cytokine signaling. One group of these IL-18 dependent chemokines is known to coordinate the recruitment of neutrophils to sites of infection (Fig 2a, depicted in green). The accumulation of neutrophils in the infected mucosa is a hallmark of *S*.Tm-induced tissue inflammation [16, 33] and represents a key line of pathogen defense in the cecum lumen, the cecum tissue as well as at systemic sites at later stages of the infection [34–39]. However, their function in eliciting mucosal inflammation had remained unclear. We speculated that reduced neutrophil recruitment in IL-18-deficient mice could explain their delayed onset of mucosal pathology.

Thus, we have analyzed the role of neutrophils in the initial phase of the infection. *S*.Tm-mediated induction of two candidate chemokines (CXCL1 and CXCL2) as well as their reduced induction in IL-18-deficient animals was confirmed by qRT-PCR (Fig 2b). In line with this expression pattern, we observed a significant accumulation of Ly-6G^+^CD11b^+^CD45^+^ cells (i.e. neutrophils) in the infected mucosal tissue compared to naïve animals (12h p.i., 5x10^7^ CFU *S*.Tm; Fig 2c and 2d). In contrast, the lamina propria of infected *Il18* ^*-/-*^ mice featured significantly decreased amounts of Ly-6G^+^CD11b^+^CD45^+^ cells compared to their littermate controls (Fig 2e and 2f), although recruitment was not completely blunted. This verified that IL-18 affects neutrophil recruitment to the infected cecum mucosa already early in *S*.Tm infection, presumably via the induction of neutrophil-recruiting chemokines.

In order to determine, if the recruited neutrophils functionally contribute to the onset of mucosal inflammation, we depleted neutrophils using a combination of anti-G-CSF (0.4mg/kg) and anti-Ly-6G (6mg/kg) antibodies. Mice were infected with *S*.Tm for 12h and enteropathy was assessed by pathoscoring. All mice showed equivalent levels of cecum luminal *S*.Tm colonization (S2a Fig). Interestingly, neutrophil depletion did not significantly alter the mucosal pathology (Fig 2g and 2h, S2b Fig). In spite of the blunted neutrophilic influx into the tissue and lumen, tissue pathology was comparable between neutrophil-depleted animals and non-depleted controls. As neutrophils have been described to limit *S*.Tm loads in the cecum lumen and at systemic sites at later stages of the infection [38, 39], we hypothesized that they might still limit *S*.Tm tissue loads at 12h p.i.. To address this hypothesis, we infected mice depleted of neutrophils and non-depleted controls with *S*.Tm carrying a reporter expressing GFP from a SPI-2 promoter once the pathogen has invaded host cells (*pssaG*-GFPmut2; [31]) and enumerated the GFP-positive *S*.Tm in the epithelial layer. Surprisingly, we could observe no difference in *S*.Tm tissue loads between mice depleted of neutrophils and WT animals (S2c Fig). In conclusion, our data identify an early recruitment of neutrophils to the infected LP, which is partially dependent on the presence of IL-18. However, during this early state of infection, neutrophils do not seem contribute to the onset of tissue pathology or the control of *S*.Tm tissue-loads. We conclude that reduced neutrophil counts cannot explain the delayed tissue pathology of the IL-18-deficient animals that we had observed in the experiments, above (Fig 1).

### IL-18 modulates the mounting of gut inflammation by stimulating NK cell recruitment

**Fig 3:**
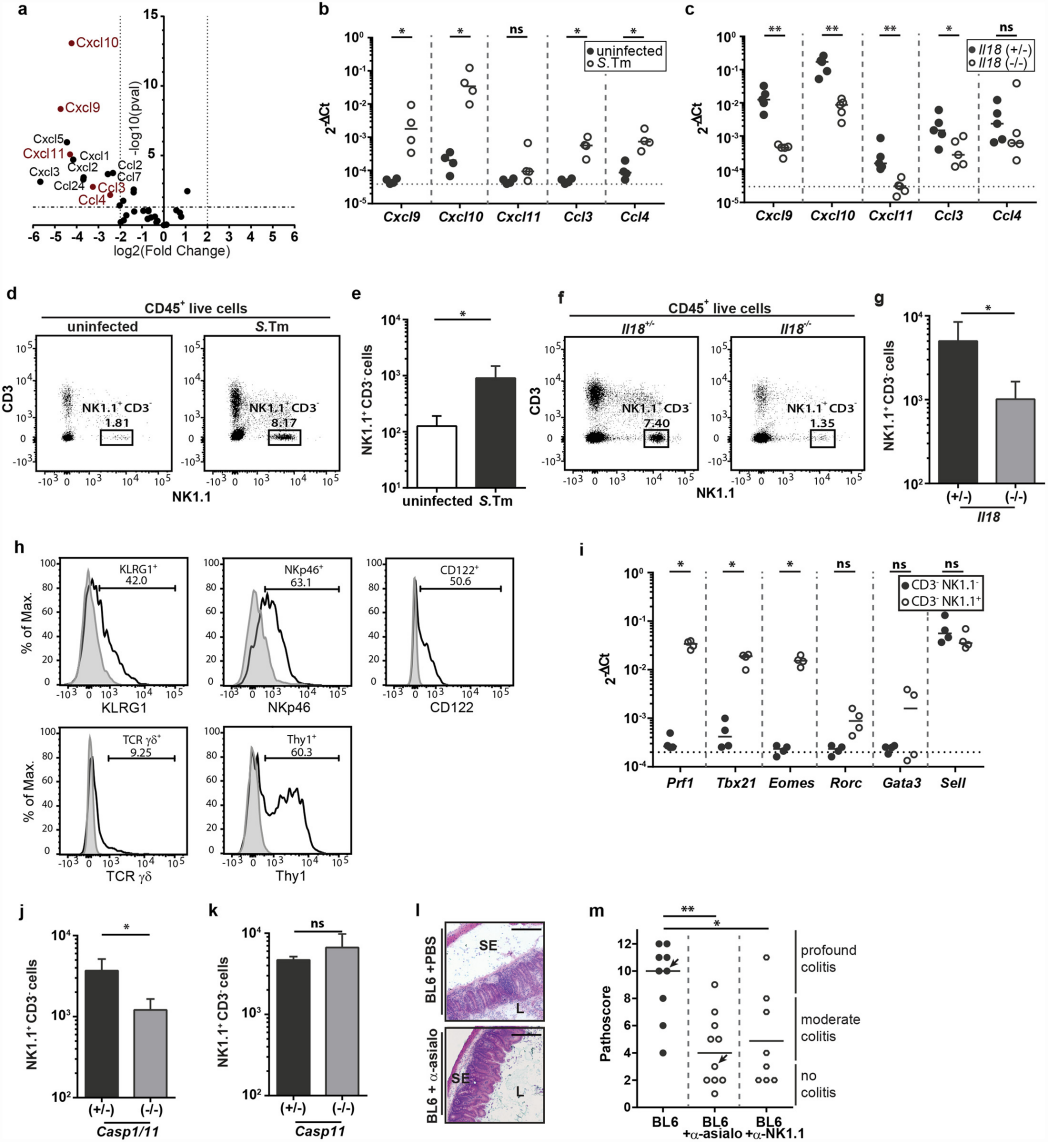
IL-18 enhances the recruitment of NK cells into the infected cecum LP thereby stimulating early cecal inflammation. (a) *Il18* ^*-/-*^ mice and littermate controls were Sm-pretreated, infected orally with 5x10^7^ CFU *S*.Tm for 12h (n = 3–4 per group). RNA-Seq was performed on RNA extracted from complete cecum tissue. RNA-Seq analysis: The Volcano plot shows the induction (log_2_ fold change) versus the -log_10_ p-value for all chemokines. Chemokines able to induce NK cell recruitment are highlighted in red. (b) C57BL/6 WT mice were Sm-pretreated and either uninfected or infected orally with 5x10^7^ CFU *S*.Tm for 12h (n = 4 per group). *Cxcl9*, *Cxcl10*, *Cxcl11*, *Ccl3* and *Ccl4* mRNA levels in whole cecum tissue were measured by RT-qPCR. Results are presented relative to the expression of *Actb*. (c) Same as (b) but comparing cecum tissues from infected *Il18* ^*-/-*^ mice vs infected littermate controls. (d and e) Flow cytometric analysis of isolated cecal LP cells from Sm-pretreated C57BL/6 WT mice, either uninfected or infected with 5x10^7^ CFU *S*.Tm (n = 5 per group). Single live cells were gated on CD45^+^ lymphocytes. (d) Representative dot plots and (e) quantification of NK1.1^+^ CD3^-^ cells. (f and g) Flow cytometric analysis of isolated cecal LP cells from *Il18* ^-/-^ mice and littermates, Sm-pretreated and orally infected with 5x10^7^ CFU *S*.Tm for 12h (n = 5–6 per group). Single live cells were gated on CD45^+^ lymphocytes. (f) Representative dot plots and (g) quantification of NK1.1^+^ CD3^-^ cells. (h) C57BL/6 mice were infected for 12h with 5x10^7^ CFU *S*.Tm and isolated cecal LP cells of two mice were pooled for staining and isotype control staining; data are shown for one out of three independent experiments. CD45^+^ NK1.1^+^ CD3^-^ cells were characterized according to their surface expression of KLRG1, NKp46, CD122, TCRγδ and Thy1. (i) C57BL/6 mice were infected for 18h with 5x10^7^ CFU *S*.Tm. Isolated cecal LP cells of two mice each were pooled for fluorescence activated cell sorting. Live CD45^+^ CD3^-^ cells were further sorted according to their expression of NK1.1. *Tbx21*, *Eomes*, *Rorc*, *Gata3*, *Prf1 and Sell* transcripts were analyzed by RT-qPCR. Results are presented relative to the expression of *Actb*. (j and k) Flow cytometric analysis of isolated cecal LP cells from Sm-pretreated (j) Casp1/11^-/-^ or (k) Casp11^-/-^ mice. Single live cells were gated on CD45^+^ lymphocytes and NK1.1^+^ CD3^-^ cells were quantified. (l and m) C57BL/6 WT mice were injected intraperitoneally with anti-asialo GM1 antiserum (50μL antiserum/mouse; three consecutive days), anti-NK1.1 (10mg/kg; 2 consecutive days) or PBS and mice were infected orally with 5x10^7^ CFU *S*.Tm for 12h (n = 8–10 per group). (l) HE-stained cryosections of anti-asialo GM1 -treated or control mice, (m) pathological score; arrows indicate mice of representative HE-stained cryosections; SE = submucosal edema, L = lumen; scale bar = 100μm. Statistical analysis was performed using the Mann-Whitney-U test (ns = not significant, * = p<0.05; ** = p<0.01).

In addition to Neutrophil recruiting chemokines, a second group of chemokines was significantly differentially expressed in our RNA-Seq dataset, which is known to coordinate the recruitment of NK cells to sites of infection (Fig 3a, depicted in red;[40]). NK cells are early effectors in the mucosal defense against several viruses, bacteria, protozoa and fungi [41]. They respond to a wide range of chemokines, are rapidly mobilized in response to danger signals and therefore quickly recruited to sites of inflammation and disease [42]. There, NK cells are activated either indirectly by cytokines or directly via the recognition of stressed and infected cells [43–45]. During systemic bacterial infections, activated NK cells can limit tissue infection and prevent systemic spread of the pathogen through direct lysis of target cells or by releasing GM-CSF, TNF and IFNγ to orchestrate further responses [41]. The secretion of such pro-inflammatory cytokines by NK cells and other early effector cells orchestrates and amplifies the local immune response, inducing a full-blown mucosal inflammation to boost pathogen elimination. Our findings provided a first hint that NK cells might be important coordinators initiating gut inflammation.

For most of those chemokines, we could confirm their *S*.Tm-mediated induction and their IL-18 dependence by qRT-PCR (Fig 3b and 3c). In line with this, the cecum mucosa of infected WT control mice featured ~10-fold higher densities of NK1.1^+^ CD3^-^ CD45^+^ cells than that of uninfected animals (12h p.i., 5x10^7^ CFU *S*.Tm; Fig 3d and 3e). Time course experiments verified the recruitment of NK1.1^+^ CD3^-^ CD45^+^ cells during the initial 12-18h p.i. (S3a Fig). In contrast, IL-18-deficient animals harbored reduced numbers of NK1.1^+^ CD3^-^ CD45^+^ cells in the infected cecal mucosa (Fig 3f and 3g). These data suggest that IL-18-dependent chemokine responses control the accumulation -and most likely also the function- of NK1.1^+^ CD3^-^ CD45^+^ cells in the cecum tissue during the initial phase of the pathogen-host interaction.

We further characterized the accumulating NK1.1^+^ CD3^-^ cells according to their surface expression of KLRG1, NKp46, CD122, TCRγδ and Thy1 (Fig 3h). Most NK1.1^+^ CD3^-^ cells were identified as KLRG1^+^ NKp46^+^ CD122^+^ Thy1^+^ TCRγδ^-^ NK1.1^+^ CD3^-^ NK cells. As expected for NK cells, this population expressed high levels of the transcription factors *Eomes* and *Tbx21* as well as *Prf-1* and *Sell* transcripts, while mRNA-levels of transcription factors Rorc and Gata3 were not significantly affected (Fig 3i).

To further verify the IL-18 function in NK cell recruitment, we performed experiments on caspase-1/11-deficient mice. As these mice produced reduced levels of mature IL-18 protein in response to mucosal infection (see Fig 1d), we reasoned that these animals should feature reduced NK cell numbers in the infected cecum tissue. 12h infection experiments with caspase-1/11-deficient animals and their littermate controls verified that this is indeed the case (Fig 3j and S3b Fig). In contrast, caspase-11-deficient mice featured equivalent mucosal NK cell numbers as their littermate controls (Fig 3k and S3c Fig). This provided further evidence supporting a link between mucosal IL-18 induction and the accumulation of NK cells during *S*.Tm infection.

In order to assess their functional importance, we depleted NK cells using an α-asialo GM1 antiserum and infected mice with *S*.Tm (5x10^7^ CFU *S*.Tm, 12h p.i.). The depletion efficiency was verified by flow cytometry (S3d Fig). Both experimental groups showed equal bacterial loads in the mLN, suggesting that initial pathogen translocation kinetics were not accelerated in the absence of NK cells. The cecal pathology was assessed by pathoscoring. Strikingly, depletion of NK cells during the *S*.Tm infection reduced levels of mucosal pathology compared to mock-depleted controls (Fig 3l and 3m, S3e Fig). Equivalent observations were made when using an α-NK1.1 antibody for cell depletion (clone PK136, 10mg/kg, i.p.; Fig 3m and S3e Fig). This antibody is less specific than the α-asialo GM1 antiserum, as it recognizes not only NK cells, but also additional NK1.1^+^ cell populations. Nonetheless, both NK cell depletion strategies recapitulate the delayed mucosal inflammation observed in IL-18-deficient mice (compare Figs 1b and 3m). These data provided a first indication that IL-18 dependent NK-cell recruitment is central for initiating the inflammatory response.

### NK cells display an enhanced migratory potential in presence of IL-18

So far, we could show that the initiation of mucosal inflammation depends on IL-18, that this leads to changes in the cellular composition of the mucosa (including increased NK cell abundance) and that NK cells are required for mounting the disease. However, it had remained unclear how NK cells are engaged in the process. At least three different mechanisms might be involved, i.e. enhanced NK-cell immigration, increased NK cell proliferation in the infected tissue and/or elevated NK-cell activation within the responding tissue.

**Fig 4:**
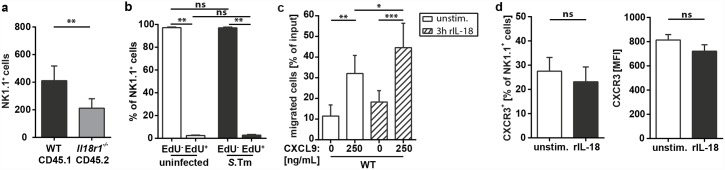
IL-18 enhances the migratory potential of NK cells. (a) Flow cytometric analysis of isolated cecal LP cells from 1:1 *Il18r1* ^-/—^CD45.2:WT-CD45.1 mixed bone marrow chimeric mice, Sm-pretreated and infected orally with 5x10^7^ CFU *S*.Tm for 12h (n = 9 per group). Single cells were gated on CD45^+^ CD3^-^ lymphocytes. Quantification of NK1.1^+^ cells from WT or *Il18r1* ^-/-^ LP cells as distinguished by their congenic markers CD45.1 (WT) and CD45.2 (*Il18r1* ^-/-^). (b) Assessment of *in vivo* cell proliferation via EdU incorporation and flow cytometric analysis of isolated LP cells from C57BL/6 mice, either uninfected or infected orally with 5x10^7^CFU *S*.Tm for 12h (n = 5–6 per group). Quantification of EdU incorporation in CD3^-^ NK1.1^+^ cells. (c) 2D Transwell migration assay of splenic NK cells isolated by MACS and stimulated for 3h in presence or absence of 100ng/mL rIL-18. Migration was analyzed towards the indicated concentrations of CXCL9 (n = 4 per group). (d) Flow cytometric analysis of CXCR3 surface expression on splenic NK cells, stimulated for 3h in presence or absence of 100ng/mL rIL-18 (n = 4 per group). Data represent the mean ± SD and statistical analyses were performed using the Mann-Whitney-U test or ordinary one-way ANOVA (ns = not significant, * = p<0.05; ** = p<0.01; *** = p<0.001).

First, we analyzed the IL-18 receptor dependency of NK cell accumulation. Here, it was important to find out if NK-cell immigration is driven by direct IL-18 signaling (via the NK-cell's IL-18 receptor) or by the NK-cell recruiting chemokines produced (by other cells) in the infected mucosa (Fig 3a–3c). It should be noted that our initial experiments in IL-18 deficient animals could not distinguish between these two mechanisms, as IL-18 deficiency also reduced tissue inflammation and chemokine production (other than IL-18). Therefore, it remained unclear whether a defective NK cell response can be directly attributed to IL-18 signaling. To address this issue, we reconstituted lethally irradiated CD45.1 WT mice with a 1:1 mixture of CD45.1 WT and CD45.2 *Il18r1* ^*-/-*^ bone marrow. These mice were infected with *S*.Tm and we analyzed NK cell numbers from both genotypes in the infected cecal LP. In this setting, we could directly compare both genotypes within the same mucosa. Compared to WT (CD45.1^+^) NK1.1^+^ cells, the *Il18r1* ^*-/-*^ (CD45.2^+^) NK1.1^+^ cells accumulated in significantly lower numbers in the infected mucosa (Fig 4a). In contrast, WT and mutant cells were present at equivalent frequencies in the blood (S4a Fig). This suggests that IL-18 directly, and not the altered inflammatory environment of the mucosa, affects the accumulation of NK cells in the mucosa during the first hours of *S*.Tm infection. Notably, the direct effect of IL-18 implies that reduced mucosal NK cell accumulation in absence of IL-18 (Fig 3d–3i) is likely attributable to both, reduced NK cell chemokine levels and a direct effect of IL-18 via the IL-18R of NK cells.

There are at least two conceivable modes of action for IL-18 in this scenario. Either it stimulates proliferation of the NK cells in the infected mucosa, thereby expanding the population *in situ*, or it enhances the migratory capacity of NK cells, thus boosting NK cell recruitment. To address the first scenario, we analyzed the proliferation of LP NK cells using an *in vivo* EdU incorporation assay. In contrast to the clear increase of NK1.1^+^ cell abundance in the infected mucosa, the fraction of EdU^+^ cells within this subset remained virtually unchanged (Fig 4b). As control, we measured in parallel the EdU incorporation in CD11b^+^ NK1.1^-^ cells, which should comprise different myeloid subsets known to proliferate in inflamed tissue [46, 47]. In contrast to the NK1.1^+^ cells, the infected mucosa featured highly increased fractions of EdU^+^ CD11b^+^ NK1.1^-^ cells (S4b Fig). This argues against an *in situ* proliferation of NK cells in response to IL-18. To verify that IL-18 has an impact on the migratory behavior of NK cells, isolated NK cells (purity ~95%, S4c Fig) were stimulated *ex vivo* with rIL-18 (100ng/mL rIL-18, 3h) and examined in 2D Transwell migration experiments using CXCL9, a classical NK cell recruiting chemokine [40]. Indeed, stimulation with IL-18 increased the migratory efficiency of NK cells, in particular at lower CXCL9 concentrations (50 or 250 ng/ml; Fig 4c and S4d Fig). This increased migratory potential was clearly dependent on IL-18 signaling, as IL-18R-deficient NK cells were unresponsive to the stimulation and showed a migration comparable to unstimulated WT NK cells (S4e Fig).

As IL-18-stimulated NK cells displayed an increased migratory potential, we examined if this can be attributed to an up-regulated surface expression of the CXCL9 receptor, CXCR3. However, rIL-18 stimulation affected neither the number of CXCR3-expressing NK cells, nor the amount of CXCR3 surface expression on stimulated NK cells (Fig 4d). This suggested that IL-18 enhances CXCL9/CXCR3 signaling downstream of the receptor (CXCR3). In summary, these data support that IL-18 increases the migratory capacity of NK cells (by engaging the NK-cell's IL-18 receptor), thereby enhancing NK cell recruitment to the infected mucosal tissue.

### Mucosal NK cells recruited in presence of IL-18 are phenotypically mature

**Fig 5:**
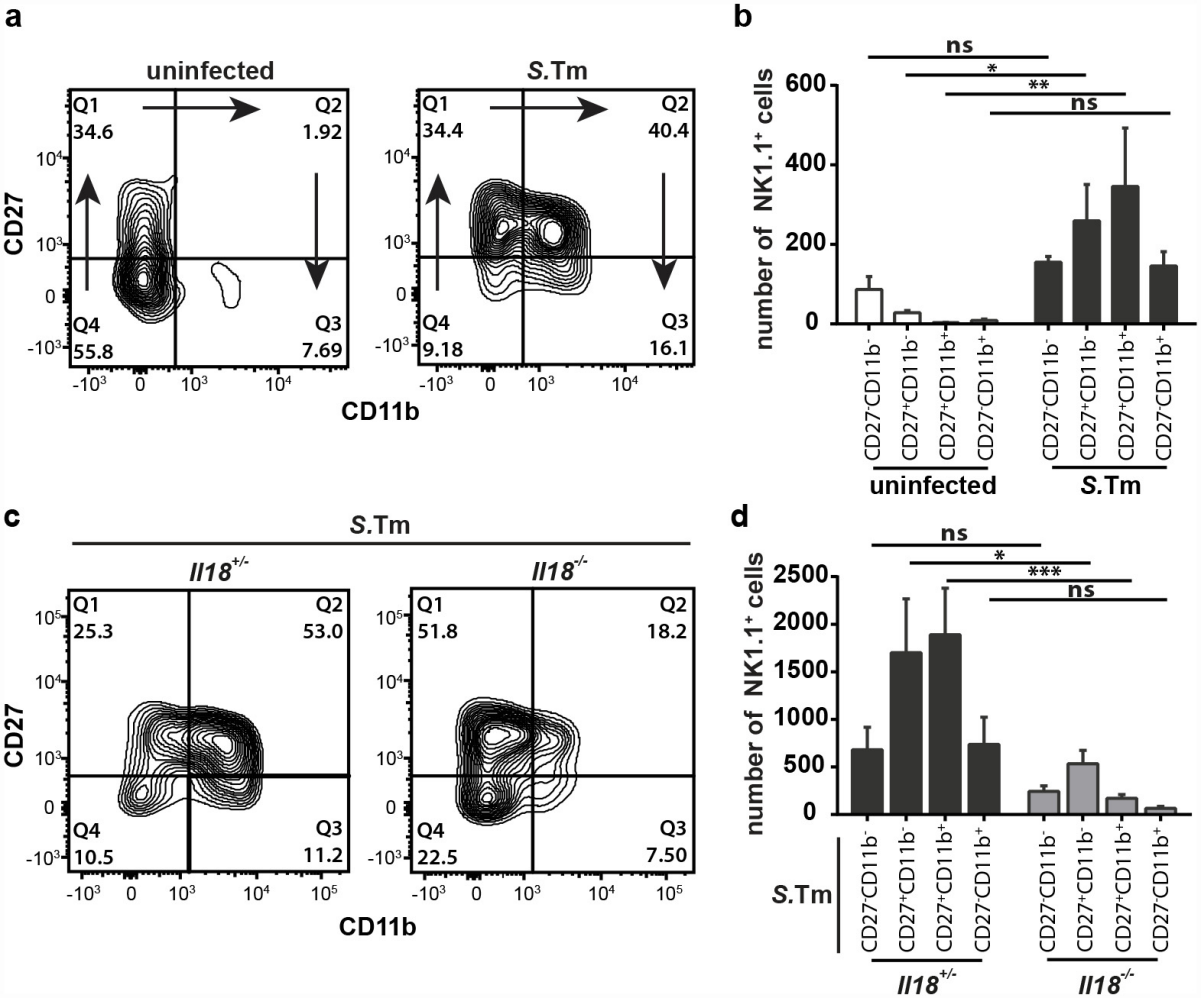
NK cells in the infected cecal mucosa of IL-18-deficient mice are phenotypically immature. (a and b) Flow cytometric analysis of isolated cecal LP cells from Sm-pretreated C57BL/6 WT mice, either uninfected or infected with 5x10^7^ CFU *S*.Tm (n = 5 per group). We gated on single live CD45^+^ CD3^-^ NK1.1^+^ cells. (a) Representative contour plots and (b) quantification of NK cell maturation stages, defined by the surface expression of CD27 and CD11b; arrows indicate the progression of NK-cell maturation. (c and d) Flow cytometric analysis of isolated cecal LP cells from *Il18* ^-/-^ mice and littermates, Sm-pretreated and orally infected with 5x10^7^ CFU *S*.Tm for 12h (n = 5–6 per group). We gated on single live CD45^+^ CD3^-^ NK1.1^+^ cells. (c) Representative contour plots and (d) quantification of NK cell maturation stages based on CD27 and CD11b surface expression. Data represent the mean ± SD and statistical analyses were performed using 2way-ANOVA with Sidak’s multiple comparison test (ns = not significant, * = p<0.05; ** = p<0.01; *** = p<0.001).

Throughout the body, tissue NK cells are featuring distinct functions and maturation stages [48]. By convention, the surface expression of CD11b and CD27 defines four maturation stages of murine NK cells [49]. These correspond to the NK cells' capacity to produce cytokines and their cytotoxic potential. Immature double negative CD27^-^ CD11b^-^ NK cells follow the maturation profile -> CD27^+^ CD11b^-^ -> CD27^+^ CD11b^+^ double positive -> CD27^-^ CD11b^+^ NK cells. Of these developmental stages, both CD11b^+^ NK cell populations are considered as *mature* as they exert typical NK cell effector functions [49–51]. The phenotype of the NK cells accumulating in the *S*.Tm infected gut mucosa remained to be established. The cecal mucosa of non-infected C57BL/6 mice harbored only a small number of NK cells and these mainly expressed immature phenotypes (CD27^-^ CD11b^-^ and CD27^+^ CD11b^-^; Fig 5a and 5b). During the first 12h of infection, CD27^+^ CD11b^+^ NK cells accumulated in the infected mucosa (Fig 5a and 5b), indicating that NK cells not only increase in abundance, but also display a higher degree of maturation. In contrast, in the infected mucosa of IL-18-deficient mice, NK cell remained scarce and mainly exhibited immature phenotypes (CD27^-^ CD11b^-^ and CD27^+^ CD11b^-^; Fig 5c and 5d). In fact, the maturation state of NK cells in infected IL-18-deficient animals resembled that of uninfected WT mice. These data suggest that IL-18 affects not only the recruitment, but also the maturation state of NK cells in the infected cecal mucosa.

### NK cell-derived cytokines do not contribute to the onset of mucosal inflammation

**Fig 6:**
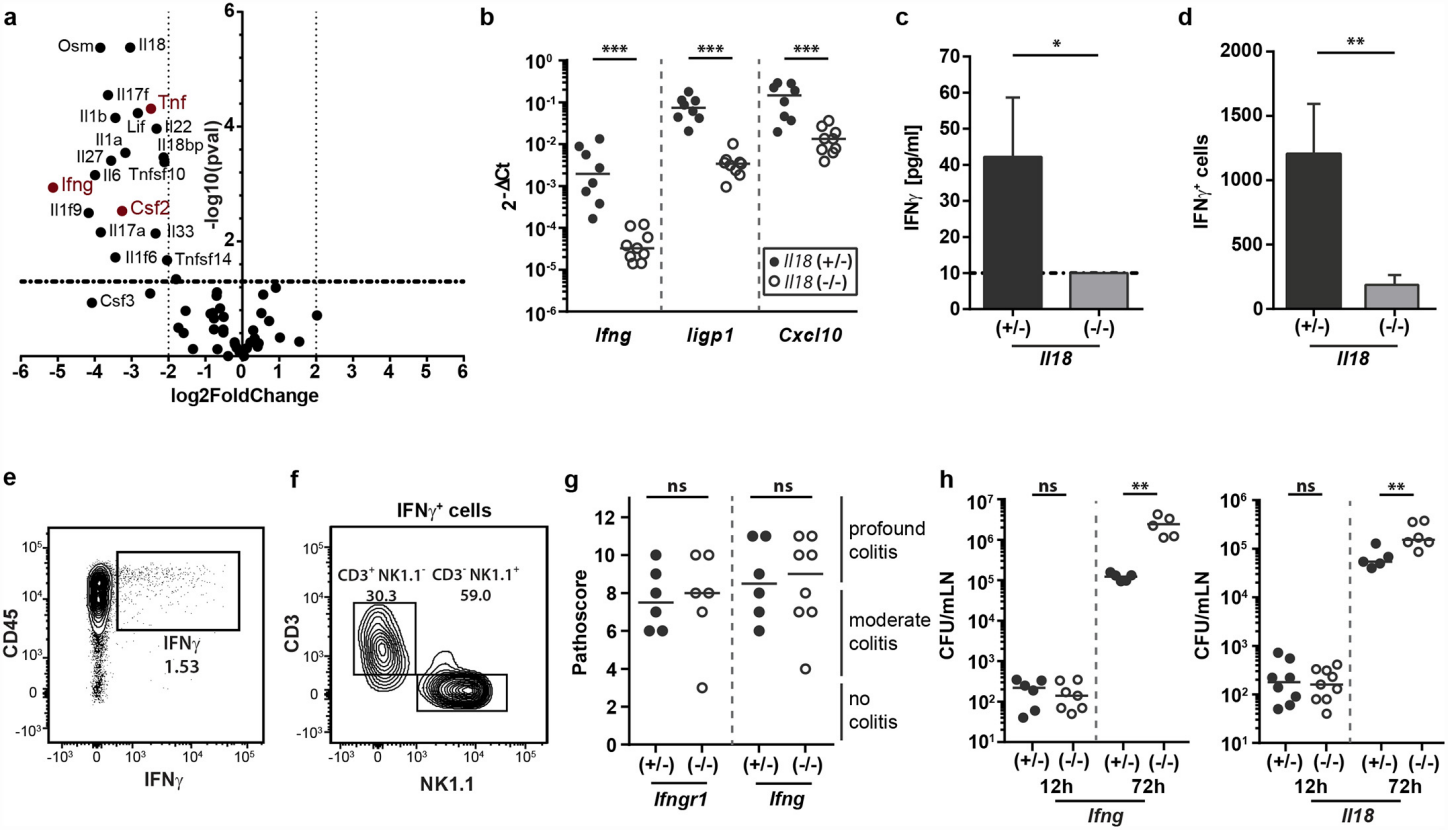
IFNγ expression by NK cells is IL-18-dependent but not required for mounting tissue inflammation during the first 12h of the infection. (a) Volcano plot of all cytokines differentially expressed in the cecal mucosa of the *Il18* ^*-/-*^ mice and littermate shown in Fig 2A. We plotted log_2_ (fold change) against -log_10_ (p-value). NK cell effector cytokines are highlighted in red. (b-d) *Il18* ^-/-^ mice and littermate controls were Sm-pretreated and infected orally with 5x10^7^ CFU *S*.Tm for 12h (b) or 18h (c-d). (b) *Ifng*, *Iigp1* and *Cxcl10* transcripts in whole cecum tissue were analyzed by RT-qPCR. Results are presented relative to the expression of *Actb* (n = 8–9 per group). (c) IFNγ protein concentration in whole cecum tissue lysates (n = 5 per group) as measured by CBA; dashed line = detection limit. (d) Quantification of IFNγ-producing cells by flow cytometric analysis of isolated cecal LP cells (n = 6 per group). (e and f) C57BL/6 mice were infected for 18h with 5x10^7^ CFU *S*.Tm and cecal LP cells were isolated for staining. Data are shown from one out of three independent experiments. (e) Representative dot plot of IFNγ-expressing cells, pre-gated on single live lymphocytes. (f) FACS-analysis of CD3 and NK1.1 surface marker expression by IFNγ^+^ cell populations. (g) *Ifng* ^-/-^, *Ifngr1* ^-/-^ and littermate controls were Sm-pretreated, infected orally with 5x10^7^ CFU *S*.Tm for 12h and pathological scores were assessed (n = 6–8 per group). (h) Mesenteric lymph node loads as determined by plating of organs from (left) *Ifng* ^-/-^ and (right) *Il18* ^-/-^ mice and their littermate controls. Mice were Sm-pretreated and infected orally with 5x10^7^ CFU *S*.Tm for 12h or 72h. Statistical analysis was performed using the Mann-Whitney-U test (ns = not significant, * = p<0.05; ** = p<0.01; *** = p<0.001).

As our previous analysis has revealed that the accumulated NK cells in the infected cecal LP are phenotypically mature, we next wanted to address the NK cell effector function, contributing to the onset of cecal inflammation. NK cells can affect defense via (at least) two different mechanisms, i.e. their cytotoxic function and the production of effector cytokines that boost antimicrobial defenses of other cell types [41, 52]. Our RNA-Seq data suggested a decreased expression of the three major NK cell-derived effector cytokines TNF, GM-CSF and IFNγ (Fig 6a, depicted in red). Therefore, we investigated the potential contribution of those three cytokines in the induction of early mucosal pathology after *S*.Tm infection. Although RNA-Seq analysis had shown a clear downregulation of GM-CSF transcripts, GM-CSF protein was not yet detectable in the cecal mucosa at 12h p.i. (S5a Fig) rendering it an unlikely candidate for promoting pathology at this initial phase of the infection. Other than GM-CSF, TNF protein levels were induced in the cecal LP by 12h p.i. and markedly reduced in IL-18-deficient mice (S5b and S5c Fig). However, flow cytometric analysis of TNF-producing cells in the cecal LP uncovered that the protein was not produced by NK cells but rather by cells from the myeloid compartment, at least at this early stage of the infection (S5d Fig). This excluded TNF as a likely NK cell effector cytokine and prompted us to focus on IFNγ.

RT PCR analyses confirmed that transcripts of *Ifng* and IFNγ-regulated genes known to be important in diverse innate and adaptive IFNγ-dependent antibacterial responses were significantly reduced in IL-18-deficient mice (*Iigp1* and *Cxcl10* shown as examples, Fig 6b). In addition, the IFNγ protein levels did not rise above the detection limit in the infected mucosa of IL-18-deficient mice (Fig 6c). Flow cytometry of cecum LP cells revealed that the absence of IFNγ is attributable to decreased populations of IFNγ-producing cells in the *Il18* knockout animals (Fig 6d) as well as to reduced IFNγ levels per cell (S5e Fig). These populations could be partially rescued by the injection of rIL-18 (120μg/kg, i.p.) into infected IL-18-deficient mice (S5f Fig). Flow cytometric analysis confirmed that the majority of IFNγ^+^ cells were CD45^+^ CD3^-^ NK1.1^+^ lymphocytes (Fig 6e and 6f) expressing CD11b, Thy1, NKp46 and Eomes (S5g Fig), and are likely identical with the NK cells identified above (see Fig 3h and 3i). In line with earlier work, some CD3-positive cells (likely T-cells) can also produce IFNγ in response to IL-18 (Fig 6f) [53, 54]. However, our NK cell depletion assays suggest that they do not affect the kinetics of mucosal inflammation. Taken together, these data support that IL-18 induces IFNγ production by NK cells during the early phase of the mucosal response to *S*.Tm infection. However, it had remained unclear, if this IFNγ is functionally required to drive the tissue inflammation observed by 12h p.i..

To address if IFNγ enhances the inflammatory pathology by 12h p.i., we infected mice deficient in IFNγ signaling (*Ifng* or *Ifngr1* knockout mice) and appropriate littermate controls with *S*.Tm. In contrast to IL-18-deficient or NK cell-depleted animals, IFNγ- and IFNγR-deficient mice showed equivalent levels of cecal pathology as the littermate controls (Fig 6g and S5h Fig, compare with Figs 1b and 2m). This indicated that a functional IFNγ response by NK cells is not required to initiate mucosal inflammation.

It should be noted that IFNγ is well-known to exert important functions limiting bacterial growth at systemic sites at later stages of typhoid-fever-like disease [55]. To verify this in our infection model, we infected IFNγ- and IL-18-deficient mice for 72h with *S*.Tm, a time point when the pathogen has spread from the mucosal tissue to systemic organs [15, 20]. Indeed, in the mesenteric lymph nodes of both, IFNγ- and IL-18-deficient mice, we detected significantly elevated *S*.Tm loads at 72h p.i. (Fig 6h). This verified that IL-18 induced IFNγ is dispensable for mounting mucosal inflammation, but important for the subsequent restriction of pathogen spread to systemic sites.

### Perforin is required to mount the mucosal inflammation

**Fig 7:**
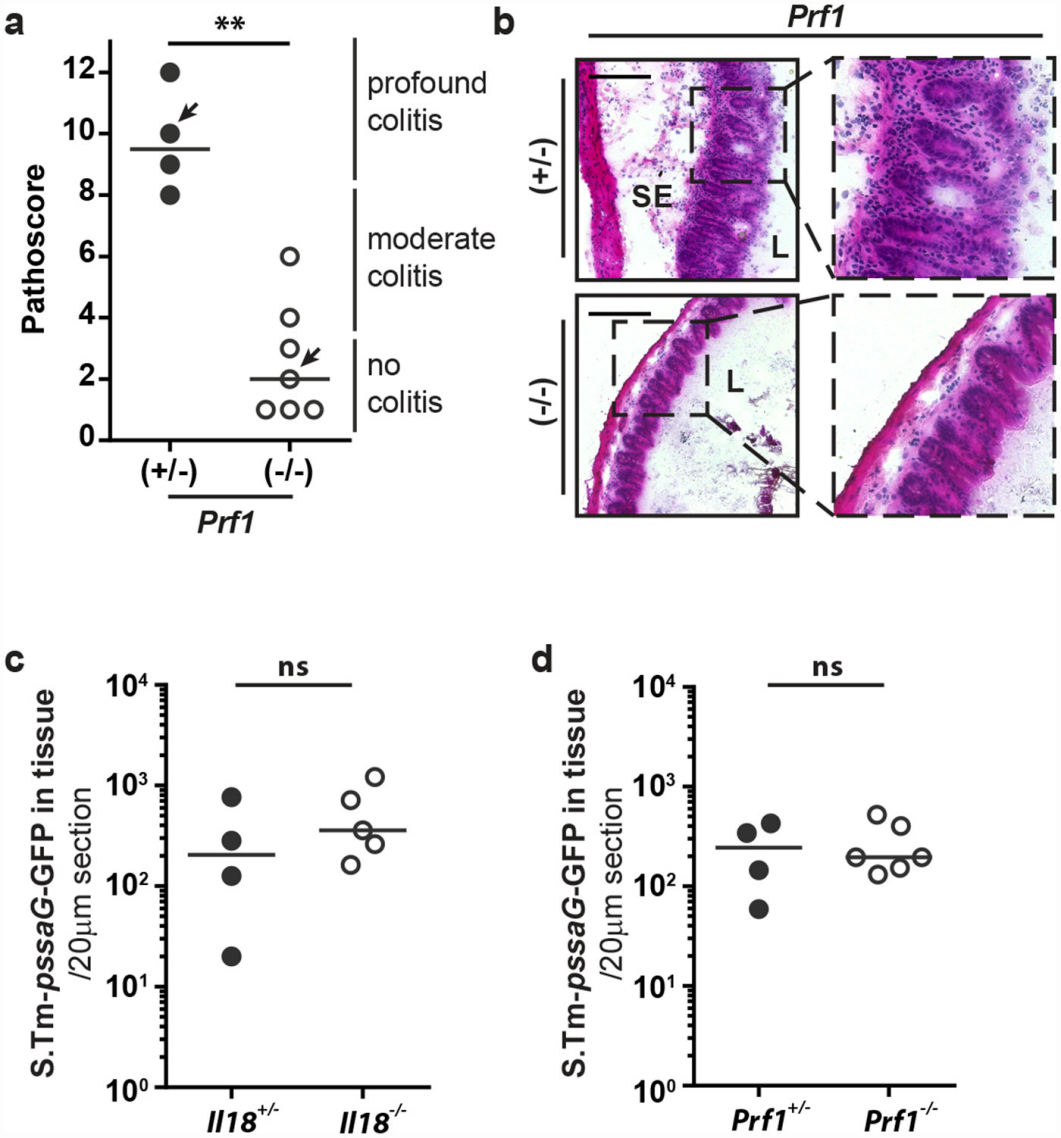
Perforin-deficient mice fail to elicit overt gut inflammation by 12h p.i. (a and b) *Prf1* ^*-/-*^ mice and littermate controls were Sm-pretreated and infected orally with 5x10^7^ CFU *S*.Tm for 12h. (a) Pathological score; arrows indicate representative mice depicted in panel b, (b) HE-stained cryosections from representative mice of each group. (c) *Il18* ^*-/-*^ mice and littermates or (d) *Prf1* ^*-/-*^ mice and littermates were Sm-pretreated and infected orally with 5x10^7^ CFU *S*.Tm-p*ssaG*-GFPmut2 for 12h (n = 4–6 per group). *S*.Tm cecum tissue counts were determined per 20μm cross-section. Statistical analysis was performed using the Mann-Whitney-U test (** = p<0.01).

Besides the production of pro-inflammatory cytokines, NK cells can exert their effector function by inducing cell death of target cells [41], either by inducing target cell apoptosis by death ligand signaling via TRAIL or FasL or by releasing cytotoxic granules, containing proteases called granzymes and perforins [56–58]. IL-18 can prime this cytotoxicity [59]. Upon release of the granules in close proximity to the target cell, perforin forms pores in the plasma membrane, enabling granzyme uptake into the target cell and subsequent induction of apoptotic cell death or osmotic cell lysis. These mechanisms are well known to eliminate virus-infected host cells [60]. A role in bacterial infections *in vivo* (i.e. liver infection by *Chromobacterium violaceum* and *Citrobacter rodentium* infection in the colon) has only recently been identified [6, 61]. Other systemic infections by intracellular pathogens (e.g. *S*.Tm, *Listeria* monocytogenes) are not affected in perforin-deficient mice, presumably due to down-regulation of inflammasome/IL-18 stimulating ligands at these sites [6, 39, 62]. However, it remained unclear if NK-cell mediated cytotoxicity might be involved in the initial phases of *S*.Tm gut infection. Based on the increased levels of mature IL-18 in the infected mucosa (Fig 1a) and the accumulation of matured NK-cells by 12h p.i., we hypothesized that this might indeed be the case. To this end, we infected perforin-deficient animals and littermate controls for 12h (5x10^7^ CFU *S*.Tm by gavage) and assessed cecal pathology. Strikingly, perforin-deficient mice showed a much lower degree of mucosal pathology than their littermate controls (Fig 7a and 7b, S6b Fig ), while luminal pathogen loads were not affected (S6a Fig). In fact, perforin-deficiency fully recapitulated the delayed mucosal pathology observed in IL-18-deficient mice and NK cell-depleted animals (compare Fig 7a to Figs 1b and 2m). This provided a first hint suggesting that perforin is an important NK cell effector mechanism that accelerates disease kinetics in the early phase of *S*.Tm infection.

### NK cells seem to be the relevant source of the perforin

NK cells are not the only effector cell type capable of inducing perforin-dependent cell death. Clearly, cytotoxic T cells could represent an alternative perforin source during infection. Therefore, we addressed a possible contribution of cytotoxic CD8^+^ T cells in the IL-18 driven mucosal immune response. However, the LP of infected *Il18* ^*-/-*^ mice (5x10^7^ CFU *S*.Tm by gavage; 12h p.i.) featured similar amounts of CD8^+^ T cells as their littermate controls (S7a–S7c Fig). In addition, depletion of CD8^+^ cells using an anti-CD8 antibody (200μg/mouse, i.p.; S7e Fig) did not significantly alter mucosal pathology upon *S*.Tm infection (5x10^7^ CFU *S*.Tm, 12h; S7f and S7g Fig). These data suggest that cytotoxic CD8^+^ T cells are not involved in early *S*.Tm-induced mucosal inflammation and suggest that other cell types like the NK-cells are the relevant source of perforin. The latter would be in line with observations from NK-cell transfer experiments (S6d–S6f Fig).

It has been shown recently that NK cells are able to control *Chromobacterium violaceum* loads in the liver and *Citrobacter rodentium* loads in the colon by perforin-dependent cytotoxicity [6, 61]. Therefore, we wanted to analyze if this mechanism of host protection is also involved in mucosal *S*.Tm infection. To this end, we infected IL-18- and Perforin-deficient animals for 12h with *S*.Tm carrying a reporter expressing GFP from a SPI-2 promoter (5x10^7^ CFU *S*.Tm-GFP; 12h) and enumerated bacterial tissue loads (Fig 7c and 7d). However, we could not observe any significant differences for tissue resident bacteria comparing *Il18* ^*-/-*^mice, *Prf* ^*-/-*^mice and littermate controls. This suggests that gut epithelial *S*.Tm loads are controlled independently of NK cell mediated killing, at least within the first 12h of the infection.

A second possible mechanism explaining how perforin-mediated cell cytotoxicity might affect disease pathology could reside in the release of soluble pro-inflammatory mediators from dying cells. ATP would be a likely mediator of such a response, as ATP is released from dying cells and a potent stimulus initiating the recruitment of inflammatory monocytes, macrophages and neutrophils [63–65]. In order to test any involvement of ATP signaling in the induction of tissue pathology, we treated infected C57BL/6 mice with the pan-P2 Receptor antagonist Suramin during the infection and assessed the degree of pathology at 12h p.i.. Suramin-treated animals did indeed feature reduced mucosal pathology (S6h–S6j Fig). This is consistent with a general involvement of ATP signaling in the onset of early pathology. As the host expresses numerous different P2 receptors on a broad variety of cell types, it remains to be verified if Suramin affects indeed the same pathway as triggered via perforin. Overall, however, these data indicate that NK-cells are a relevant source of perforin and that perforin-mediated NK cell cytotoxicity may affect disease kinetics by the release of host cellular molecules.

In summary, this work identifies a key signaling cascade in the cecal mucosa whereby *S*.Tm-induced IL-18 drives the recruitment of activated NK cells to the infected LP and suggests perforin mediated cytotoxicity as a novel mechanism eliciting gut inflammation.

## Discussion

The mucosal defense program that commences inflammation had remained incompletely understood. Here, we investigated how the naive intestinal mucosa mounts the initial response to *S*.Tm infection. This identified caspase-1-dependent IL-18 as a pivotal cytokine in the process. Our data establish IL-18-promoted migration and accumulation of mature NK cells and perforin-mediated NK cell cytotoxicity as a key axis driving mucosal inflammation.

The IL-18/NK cell perforin axis establishes a second arm of the innate immune defense that is elicited upon recognition of *S*.Tm virulence factors (i.e. flagella, TTSS-1;[13]) by the mucosal caspase-1 inflammasome. The first mechanism, expulsion of infected enterocytes, reduces the epithelial pathogen loads by about 50-100-fold by 12-18h p.i., but cannot completely clear the pathogen [12]. A complementary arm is identified in this paper. It is activated by the release of mature IL-18, which triggers pronounced cytokine and chemokine responses that lead to accumulation of matured NK cells that seem to promote inflammation via perforin. It is well established that inflammation results in a generalized antimicrobial state in the mucosa, featuring elevated numbers of phagocytic leukocytes, augmented production of antimicrobial peptides, and a boosting of adaptive immune responses [16, 33].

How does gut inflammation affect pathogen loads? Strikingly, the IL-18/perforin-axis is required for mounting gut inflammation within 12h, while *S*.Tm loads in the gut tissue and in the gut lumen remain unaltered. Thus, during these first 12h, pathogen loads seem to be mainly controlled via NLRC4-dependent (but IL-18 independent) expulsion of infected enterocytes [12, 13]. Only at later stages of the infection, macrophages, granulocytes and other parts of the innate immune defense seem to kick in and reduce pathogen tissue loads. In the gut lumen of streptomycin-pretreated mice (as used in the present study), the gut luminal pathogen loads are only affected at day 2 or later [38, 66]. In conclusion, the host's IL-18 dependent innate defenses and overt tissue inflammation seem to start controlling pathogen loads only after the initial 12h of infection.

During the first days of *S*.Tm infection, numerous cytokines are produced by the intestinal mucosa, including IL-1β and IL-18 [67, 68]. The release of mature IL-1β is induced in response to infection and it is known to increase host resistance to systemic spread during later stages of the disease [36, 69]. In contrast, IL-1β seems to have little (if any) role during the first 12-18h of the gut infection. Mice deficient in IL-1α/β signaling controlled the intraepithelial *S*.Tm load equally well as littermate controls [12] and exhibited wild-type cecum inflammation kinetics. Nonetheless, this does not formally exclude a redundant function for IL-1β in this initial phase or in other, non-redundant processes that may manifest only at later time points. IL-18 on the other hand is constitutively and highly expressed in the intestinal mucosa, especially by IECs [70–73]. It is thereby ideally positioned to mediate first-line responses to infection. Indeed, *Il18* ^*-*/-^ mice featured a delayed onset of inflammation. This was not attributable to perturbed homeostasis. Rather IL-18 was necessary for the initiation of mucosal inflammation. IL-18 levels of the infected cecal mucosa did critically affect disease kinetics, as decreasing or increasing IL-18 concentrations delayed or accelerated the mucosal response, respectively. IL-18 alone was, however, insufficient to elicit gut inflammation in the absence of stimuli from invasive wild type *S*.Tm. Thus, IL-18 must exert its function together with other signals whose nature remains to be elucidated.

To address the cellular source of Il-18, we have performed experiments in bone marrow chimeras (S8 Fig). The data suggest that IL-18 from epithelial/stromal as well as bone-marrow derived cells may play a role. This would be well in line with enterocytes and resident lamina propria cells as sources contributing to the IL-18 driven inflammatory response. As an epithelial NAIP/NLRC4/caspase1-inflammasome appears chiefly responsible for early recognition of invading *S*.Tm [12]), it seems likely that infected enterocytes are a key source of this cytokine which may be further supplemented from myeloid sources (e.g. resident macrophages and dendritic cell population; [74]).

Our data suggest that NK cells are critical effectors for the IL-18 dependent mounting of gut inflammation. IL-18 stimulated this NK cell response in at least two ways, i.e. via IL-18R dependent recruitment and by enhancing NK cell activation. It is a common theme that cytokine and chemokine responses act in concert to recruit leukocytes to sites of infection, thereby enhancing a local inflammatory response [75]. In our system of acute bacterial mucosa infection, IL-18 a) upregulated NK cell recruiting chemokines at the site of infection and b) stimulated the migratory capacity of NK cells. Yet, the underlying molecular mechanism of the enhanced migratory phenotype seen in IL-18 stimulated NK cells still needs to be elucidated. One explanation could reside in IL-18-mediated regulation of NK cell surface receptor expression [76]. However, our data exclude a direct effect of IL-18 on the chemokine receptor CXCR3 expression. Nevertheless, one could envision a synergy of cytokine and chemokine signaling, where the priming stimulus of the cytokine amplifies the downstream signaling events of the chemokine receptor complex, leading to increased sensitivity and an enhanced migratory capacity of the stimulated cell [77–80]. In particular the increased migratory potential at lower chemokine concentrations may indicate that is indeed the case [77].

Besides an increased NK cell accumulation, we observed a higher maturation state of mucosal NK cells in presence of IL-18, which is in line with previous findings addressing IL-18 function during NK cell responses upon viral infections [81]. The experimental design could not distinguish between an IL-18 dependent recruitment of pre-activated NK cells and an IL-18 induced *in situ* maturation of recruited NK cells. In any case, mature CD11b^+^ NK cells are generally associated with increased cytotoxicity and cytokine production [49, 50]. Indeed, IFNγ production by NK cells was reduced to background levels in the absence of IL-18. If also other NK cell activating cytokines (especially IL-12; [82, 83]) may synergize with IL-18 in IFNγ production by NK cells [84] during early mucosal *S*.Tm infection, remains to be established.

Surprisingly, early mucosal inflammation developed independent of IFNγ. This is in contrast to the important role of IFNγ during other bacterial gut infections as well as in later stages of *S*.Tm infection, where pathogen resistance, colitis and systemic pathogen restriction are coupled to a functional IFNγ response [85–87].

How do NK-cells promote the gut inflammation? Our data implicate a functional NK cell Perforin response in the elicitation of cecal inflammation during the early mucosal phase of the disease. NK cell mediated cytotoxicity is a well-known effector mechanism in anti-viral defense [88]. Recent evidence suggests that NK cells can also limit the susceptibility to *C*. *rodentium* infection as well as systemic loads of some intracellular bacteria via Perforin-dependent effector mechanisms [6, 61, 89]. However, the control of systemic *S*.Tm infection (at least at 12h p.i.) does not require perforin, whereas *Chromobacterium violaceum* infection is efficiently limited [6]. Similarly, Perforin-mediated NK cell cytotoxicity does not significantly alter mucosal *S*.Tm loads (at least at 12h p.i.). Much rather, it seems to promote the induction of mucosal inflammation. Of note, as shown by the NK cell transfer into *Rag* ^*-/-*^ *γc* ^*-/-*^animals, NK cells alone may not able to fully restore mucosal pathology (i.e. to WT C57/BL6 levels). This suggests that NK cells are necessary but may alone not be sufficient to induce full-blown inflammation in response to infection. Other cell types may also contribute to the process. First preliminary results might suggest that Perforin-mediated killing is able to enhance the inflammatory state of the cecal mucosa by inducing the release of soluble pro-inflammatory mediators like ATP from dying cells. This in turn could lead to the recruitment of other effector cells, like inflammatory monocytes, macrophages or neutrophils, to the infected mucosa [63–65] and subsequently induce mucosal tissue pathology. In addition to this ATP-dependent recruitment, previous studies have described a role for IL-18 in the recruitment of inflammatory monocytes to the infected LP via the induction of chemokine production from NK cells [90]. Moreover, we and others could show that IL-18 is able to stimulate the recruitment and activation of neutrophils [91, 92], a hallmark cell type in the infected mucosa with prominent effects during *S*.Tm infection [16, 38, 93–95]. Although our data suggests that neutrophils alone are not the driving force for early mucosal inflammation, they could play a redundant role in combination with other effector cells in the amplification of mucosal pathology. The IL-18/NK cell perforin axis may thus coordinate the recruitment and activity of a whole range of antimicrobial leukocyte responses. A detailed molecular analysis of how Perforin-mediated NK cell cytotoxicity fuels gut inflammation is an important topic for future work.

Is the IL-18/NK cell/perforin axis of general importance for initiating gut mucosal defense? So far, only few studies have focused on the initiating events and none has explored the entire mechanism. Nevertheless, there is accumulating circumstantial evidence suggesting a broader relevance. The importance for the NAIP/NLRC4/caspase-1 inflammasome in mucosal defense has been observed in *S*.Tm and *Citrobacter rodentium* infection models [11, 12]. Pro-IL-18 is highly expressed under steady state conditions in the intestinal mucosa and therefore a likely source of mature IL-18 during the first hours of pathogen attack. However, it had remained unclear how IL-18 switches the mucosa to inflammation. In DSS models, the gut inflammation is affected by IL-18. However, depending on the experimental protocols and the time points analyzed, IL-18 has enhancing or ameliorating effects [71, 96, 97]. This suggests a role for IL-18 in establishing inflammation or in regulation of the steady state defense, but does not answer if NK cell perforin responses are involved. *Toxoplasma gondii* gut infection has also provided evidence for the role of IL-18 in the mucosal response [98, 99]. In this model, IL-18 produced by bone-marrow-derived cells was critical for mucosal pathology at days 3–5 of infection and the disease was ameliorated by daily application of the IL-18 inhibitor IL-18BP [98]. However, due to the long time required to establish overt disease pathology, one cannot discern primary inductive mechanisms from tonic, multipronged effects on the global tissue responsiveness. The *S*.Tm infection data described here is the first evidence implicating perforin in mounting mucosal inflammation (which will likely help to control pathogen loads at later phases of the pathogen-host interaction). This would be in line with recent evidence from systemic *Chromobacterium violaceum* infection and colonic *Citrobacter rodentium* infection that implicated IL-18 elicited NK cell perforin as an essential effector mechanism limiting pathogen loads [6, 61]. Thus, bacterial pathogens other than *S*.Tm are also affected by the IL-18/NK cell perforin axis. Overall, the available evidence suggests that the caspase-1 elicited IL-18/NK cell perforin responses may be of general importance for coordinating defenses, including the initial stages of microbial gut infection.

In conclusion, our findings provide important new insights into the mounting of mucosal inflammation in the infected gut. In particular, they reveal the caspase-1/IL-18/NK cell axis as a central regulator of the initial disease kinetics and demonstrate the role of activated NK cell accumulation. These activated NK cells seem to promote inflammation via perforin-mediated cytotoxicity. It seems likely that this defense axis is of relevance for other enteropathogenic infections and that it coordinates additional branches of the mucosal defense. Our findings are an important step towards deciphering the multi-layered responses that protect the intestinal mucosa against microbial attack.

## Materials and Methods

### *Salmonella* Typhimurium strains and culture conditions

*Salmonella* Typhimurium SL1344 (SB300, SmR [100]) was used as WT. *S*.Tm-p*ssaG*-GFPmut2 has been previously used (e.g. [101]), *S*.Tm^avir^ (M557, *ΔinvG; sseD*::*aphT* [31]) is an isogenic derivative of SL1344. For infections, the bacteria were grown in LB/0.3M NaCl for 12h, subcultured at a 1:20 dilution for 4h, spun down and resuspended in PBS, pH 7.4.

### Mouse lines

All mice were maintained as specific pathogen-free in individually ventilated cages at the Rodent Center RCHCI (ETH Zürich) or the ETH Phenomics Center EPIC (ETH Zürich). C57BL/6*Ptprc* ^*b*^ mice (congenic marker Ly5.2^+^) originating from Charles River (Sulzfeld, Germany) were used as wild-type mice. For generation of bone marrow chimeras, B6.SJL-*Ptprc* ^*a*^ *Pepc* ^*b*^ mice (congenic marker Ly5.1^+^) were used as wild type. Knockout mouse lines *Casp1/11* ^*-/-*^ (B6.129S2-*Casp1* ^tm1Sesh^ [102]), *Casp11* ^*-/-*^ (B6.*Casp11* ^tm1^ [103]) *Il1ab* ^*-/-*^ (B6.D-*IL1a* ^*tm1Yiw*^ */IL1b* ^*tm1Yiw*^ [104]), *Il18* ^*-/-*^ (B6.129P2-*Il18* ^tm1Aki^ [105]), *Il18r1* ^*-/-*^ (B6.129P2-*Il18r1* ^tm1Aki^ [106]), *Ifng* ^*-/-*^ (B6.129S7-*Ifng* ^tm1Ts^ [107]), *Ifngr1* ^*-/-*^ (B6.129S7-*Ifngr1* ^tm1Agt^ [108]), *Prf1* ^*-/-*^ (C57BL/6-*Prf1* ^*tm1Sdz*^ [56]) and Rag2^-/-^γc^-/-^ (C57BL/6-*Rag2* ^*tm1Fwa*^.*Il2rg* ^*tm1Cgn*^[109, 110]*)* were all of C57BL/6 background. For experimentation, wild type (+/+), heterozygous (+/-) and homozygous knockout (-/-) littermates were obtained by backcrossing into C57BL/6 and genotypes were verified by PCR.

### Mice and infections

*S*.Tm infections were performed as described previously [15]. In brief, 7–12 week old mice were pretreated with 25mg/animal streptomycin sulfate (Applichem) by gavage. 24 h later mice were infected with 5×10^7^ CFU *S*.Tm by gavage. Infections were performed for 6h, 8h, 12h, 18h, 36h or 72h, as indicated. Bacterial loads in gut luminal content, mLN, spleen and liver were determined by plating. For *in vivo* treatment with murine rIL-18, mice were injected intraperitoneally with a single dose of murine r-IL18 (120μg/kg, MBL) at the time of infection. For in vivo neutralization of IL-18, mice received intraperitoneal injections of human r-IL18BP (2mg/kg, Life Technologies Europe) at the time of infection. NK1.1^+^ cells were depleted by intraperitoneal injection of anti-NK1.1 (10mg/kg, clone PK136, BioXCell) on two consecutive days, starting one day prior to infection. In addition, NK cells were depleted by intraperitoneal injection of anti-asialo GM1 antiserum (50uL/mouse; Wako); mice were injected 3 times in total, starting 3 days prior to infection. Neutrophils were depleted by a daily intraperitoneal injection of anti-G-CSF (0.4mg/kg, clone 67604, R&D Systems (Abingdon, UK)) starting one day prior to infection in combination with a single dose of anti-Ly6G (6mg/kg, clone 1A8, BioXCell) at one day prior to infection. CD8^+^ cells were depleted by intraperitoneal injection of anti-CD8 (8mg/kg, clone, BioXCell) 3 days and 1 day prior to infection. For adoptive transfer experiments, splenic NK cells from WT BL6 or Prf1^-/-^ mice were isolated by MACS using the murine NK cell isolation Kit II. Recipient Rag2^-/-^γc^-/-^ animals received 5x10^5^ purified NK cells by i.v. injection, infections were conducted 7 days post transfer.

### Generation of bone marrow chimeras

Generation of bone marrow chimeras has been described before [111]. Briefly, *Il18* ^*-/-*^, *Il18r1* ^*-/-*^ Ly5.2 and C57BL/6 Ly5.1 donor mice were euthanized and bone marrow was extracted from tibia and femur. Recipient mice (C57BL/6 Ly5.1 or *Il18* ^*-/-*^) were γ-irradiated (1000rad) and reconstituted intravenously with 5x10^6^ WT, 5x10^6^ *Il18* ^-/-^ or a 1:1 mixture of 2.5x10^6^ *Il18r1* ^*-/-*^ Ly5.2 and 2.5x10^6^ C57BL/6 Ly5.1 bone marrow cells. Mice were kept on Borgal (Veterinaria AG) for 3 weeks and were infected 8 weeks after reconstitution. Reconstitution efficiency was controlled by flow cytometry (Ly5.1/CD45.1, Ly5.2/CD45.2 staining) on LP and blood cells.

### Histopathology

Cecum tissue was frozen in OCT (comp) and stored at -80°C. 5μm cryosections were mounted on glass slides, air-dried and stained with hematoxylin and eosin. Histopathology was evaluated in a blinded manner as described previously [15], scoring submucosal edema, epithelial integrity, goblet cell number and polymorphonuclear leukocyte infiltration, resulting in a total pathological score between 0 (uninflamed) and 13 (maximally inflamed).

### Lamina propria cell isolation, staining and flow cytometry

Mice were sacrificed and the cecum was opened longitudinally and washed in ice cold PBS to remove the remaining cecal content. In order to dislodge the epithelial cells, the cecum tissue was cut into small pieces, and placed in two subsequent rounds into PBS containing 5mM EDTA, 15mM HEPES and 10% FCS, incubated for 20min at 37°C, and shaken mildly. Samples were washed in RPMI supplemented with 30% FCS and digested for 1h in RPMI containing 1mg/mL Collagenase VIII (Sigma) and 0.2mg/mL DNase I (Roche). Cells were filtered through a 70μm cell strainer and rinsed in RPMI. Isolated cells were loaded onto a NycoPrep 1.077 matrix (Progen) and centrifuged for 30 min at 400g. Cells were collected from the interface and washed in RPMI. Cell suspensions were stained in PBS containing 1% FCS and 0.02% sodium azide. For intracellular cytokine staining, isolated lamina propria cells were incubated for 5h in RPMI containing 5% FCS and 10μg/mL BrefeldinA (Sigma) at 37°C/5%CO_2_. After surface staining, cells were fixed in fix/perm solution (eBioscience) for 30min at 4°C and stained intracellular in wash/permeabilization buffer (eBioscience). Antibodies were either from Biolegend, i.e. CD45 (clone 30-F11), CD3 (clone 17A2), CD4 (clone GK1.5), CD8α (clone 53–6.8), CD8β (clone YTS156.7.7), CD27 (clone LG3.A10), IFNγ (clone XMG1.2), CD11b (clone M1/70), CD90.2 (clone 30-H12), NKp46 (clone 29A1.4), CD45.1 (clone A20), CD45.2 (clone 104), TCRγδ (clone GL3), CD122 (clone TM-β1), KLRG1 (clone 2F1/KLRG1), Eomes (clone Dan11mag) or from BD Biosciences i.e. NK1.1 (clone PK136) and TCRb (clone H57-597). For detection of 5-ethynyl-2’-deoxyuridine (EdU) incorporation, the Click-iT EdU Alexa Fluor 488 Flow Cytometry Assay Kit (Invitrogen) was used. Data were acquired on a LSRII (BD Biosciences) and analyzed using FlowJo software (TreeStar). Cell sorting was performed on a FACS Aria III (BD Biosciences) and equal amounts of CD45^+^ CD3^-^ NK1.1^+^ and CD45^+^ CD3^-^ NK1.1^-^ cells were collected separately in two flow tubes.

### Assessment of in vivo proliferation via EdU incorporation

In vivo NK cell proliferation was measured by intraperitoneal injection of EdU (400μg/mouse) 12h prior to sacrifice. Single cell suspensions were obtained from cecal tissue by cecal lamina propria isolation as described above. Incorporated EdU was detected by fluorescent-azide coupling reaction according to the manufacturer’s protocol (Invitrogen) and analyzed by flow cytometry.

### 2D Transwell migration

NK cells were isolated from spleens of naïve C57BL/6 mice by MACS using the murine NK cell isolation Kit II. Isolated spleenocytes were cultured for 3h in presence or absence of 100ng/mL rIL-18 and cell migration was assessed by the 24-well Transwell Systems and polycarbonate filters with a pore size of 5μm (Corning Costar). Briefly, 1x10^5^ cells were allowed to migrate for 3h to the lower compartment containing different concentrations of CXCL9. Migrated cells were harvested and cell numbers were determined by flow cytometry.

### Confocal microscopy

Cecum tissue was fixed in 4% paraformaldehyde/4% sucrose, saturated in PBS/20% sucrose, embedded in optimum cutting temperature medium (Tissue-Tek), flash-frozen in liquid nitrogen, and stored at −80°C; 20 μm cryosections were air-dried, rehydrated with PBS, permeabilized (PBS/0.5% Triton X-100), and blocked (PBS/10% Normal Goat Serum). Antibody stainings included anti-ICAM-1/CD54 (clone 3E2, Becton Dickinson), anti-CD18 (clone M18-2, Biolegend), appropriate secondary reagents, AlexaFluor647-conjugated phalloidin (Molecular Probes), and DAPI (Sigma Aldrich). Samples were mounted with Mowiol (Calbiochem). A Zeiss Axiovert 200 m microscope with 10×–100× objectives, a spinning disc confocal laser unit (Visitron), and two Evolve 512 EMCCD cameras (Photometrics) were used for imaging. Postcapture processing and analysis used the Visiview (Visitron) and Image J ×64. For quantification of *S*.Tm in the epithelium, 20 μm cross-sections were stained for ICAM-1, phalloidin, and DAPI and intracellular *S*.Tm-GFP^+^ were manually enumerated blindly in six to nine nonconsecutive sections/mouse. All data represent averages/section.

### RT-qPCR

For analysis of mRNA expression, total RNA was extracted from homogenized cecum tissue using the RNeasy Mini Kit (Qiagen). Total RNA from sorted cells was extracted using the RNeasy Micro Kit (Quiagen). For reverse transcription, 1μg total RNA was transcribed using the RT^2^ HT First Strand Kit (Qiagen). RT-qPCR was performed using Custom RT^2^ Profiler Arrays (Qiagen) or RT^2^ qPCR Primer Assays (Qiagen) with RT^2^ SYBR Green ROX FAST (Qiagen) on an Applied Biosystems 7900 HT Fast Real-Time PCR Cycler. Relative mRNA expression was calculated using the ΔC_t_ method, using *Actb* as reference gene [112].

### Cytokine measurement

Cecum tissue was washed in ice-cold PBS to remove remaining luminal content. The tissue was homogenized in PBS containing 0.5% Tergitol and lysates were cleared by centrifugation. IL-1β and IFN-γ concentrations were determined using the respective Cytometric Bead Array Mouse Flex Sets (BD Biosciences) with the CBA Mouse/Rat Soluble Protein Master Buffer Kit (BD Biosciences). IL-18 concentrations were measured by employing the IL-18 ELISA Kit (MBL) according to the manufacturer's protocol.

### Ethical statement

All animal experiments were subject to the Swiss animal protection law (TschG) and therefore reviewed independently by a dedicated cantonal committee (Tierversuchskommission) and approved by the "Kantonales Veterinäramt, Zürich" (licenses 223/2010 and 222/2013).

## Supporting Information

S1 Fig IL-18 influences cecum pathology during the early course of infection. (a) C57BL/6 WT mice were Sm-pretreated and either uninfected or infected orally with 5x10^7^ CFU *S*.Tm for 12h (n = 7 per group). *Il1b* and *Il18* mRNA levels in whole cecum tissue were measured by RT-qPCR. Results are presented relative to the expression of *Actb*. (b and c) *Il1ab* ^*-/-*^ and *Il18* ^*-/-*^ mice and littermate controls were Sm-pretreated and infected orally with 5x10^7^ CFU *S*.Tm for 12h (n = 6–9 per group). (b) *S*.Tm loads in cecum luminal contents and (c) parameters of cecal pathology (grey bar- submucosal edema; dotted bar- goblet cells; white bar- PMN infiltrates; striped bar- tissue integrity). (d-f) *Il18* ^-/-^ mice and littermates were Sm-pretreated and infected orally with 5x10^7^ CFU *S*.Tm for the indicated time points (n = 5–9 per group). (d) IL-18 protein levels in whole cecum tissue lysates from *Il18* littermates (n = 5–6 per group); dashed line indicates detection limit. (e) *S*.Tm load in cecum luminal content and (f) pathological score. Note that 12h data are replotted from panel 1b and S1b. (g) *Casp1/11* ^*-/-*^ and *Casp11* ^*-/-*^ mice and littermate controls were Sm-pretreated, infected orally with 5x10^7^ CFU *S*.Tm for 12h (n = 5–7 per group) and *S*.Tm load in cecum luminal content was assessed. (h) C57BL/6 WT mice were Sm-pretreated, injected intraperitoneally with rIL-18BP or PBS, infected orally with 5x107 CFU *S*.Tm for 12h (n = 5 per group) and S.Tm load in cecum luminal content was assessed. (i) C57BL/6 WT mice were Sm-pretreated, injected intraperitoneally with rIL18 or PBS, infected orally with 5x107 CFU *S*.Tm for 8h (n = 9 per group) and cecum luminal content was assessed. (j and k) C57BL/6 WT mice were Sm-pretreated, injected intraperitoneally with rIL18 or PBS and infected orally with 1x10^10^ CFU *S*.Tm^avir^ for 12h (n = 5 per group). (j) *S*.Tm^avir^ load in luminal content, (k) pathological score. Statistical analyses were performed using the Mann-Whitney-U test or 1way-ANOVA with Sidak's multiple comparison test (ns = not significant, * = p<0.05; ** = p<0.01; *** = p<0.001).
(TIF)

S2 Fig Neutrophil depletion does not influence early cecum pathology upon S.Tm infection. (a and b) C57BL/6 WT mice were injected intraperitoneally with anti-G-CSF (0.4mg/kg; two consecutive days) and anti-Ly-6G (6mg/kg; two days prior to infection) or PBS. Mice were Sm-pretreated and infected orally with 5x10^7^ CFU *S*.Tm for 12h (n = 6 per group). (a) S.Tm loads in cecum luminal content and (b) parameters of cecal pathology (grey bar- submucosal edema; dotted bar- goblet cells; white bar- PMN infiltrates; striped bar- tissue integrity). (c) C57BL/6 WT mice were injected intraperitoneally with anti-G-CSF (0.4mg/kg; two consecutive days) and anti-Ly-6G (6mg/kg; two days prior to infection) or PBS. Mice were Sm-pretreated, infected orally with 5x10^7^ CFU *S*.Tm-p*ssaG*-GFPmut2 for 12h (n = 4 per group) and *S*.Tm cecum tissue counts were determined per 20μm cross-section. Statistical analysis was performed using the Mann-Whitney-U test (ns = not significant).
(TIF)

S3 Fig NK1.1^+^ CD3^-^ cell counts in the infected LP of WT, Casp-1/11- and Casp-11-deficient mice. (a) Flow cytometric analysis of isolated cecal LP cells from Sm-pretreated C57BL/6 WT mice, either uninfected or infected with 5x10^7^ CFU *S*.Tm for the indicated time points (n = 4–5 per group). Single live cells were gated on CD45^+^ lymphocytes and NK1.1^+^ CD3^-^ cells were quantified. (b and c) Flow cytometric analysis of isolated cecal LP cells from Sm-pretreated (b) Casp1/11^-/-^ or (c) Casp11^-/-^ mice. Single live CD45^+^ lymphocytes were gated on CD45^+^ NK1.1^+^ CD3^-^ cells, shown are representative dot plots. (d) C57BL/6 WT mice were injected intraperitoneally with anti-asialo GM1 antiserum or PBS and mice were infected orally with 5x10^7^ CFU *S*.Tm for 12h (n = 10 per group). Depletion efficiency of NK1.1^+^ cells in (left) cecum and (right) blood. (e) C57BL/6 WT mice were injected intraperitoneally with anti-asialo GM1 antiserum (50μL antiserum/mouse; three consecutive days), anti-NK1.1 (10mg/kg; 2 consecutive days) or PBS and were infected orally with 5x10^7^ CFU *S*.Tm for 12h (n = 8–10 per group). Parameters of cecal pathology were assessed (grey bar- submucosal edema; dotted bar- goblet cells; white bar- PMN infiltrates; striped bar- tissue integrity). Data represent the mean ± SD and statistical analyses were performed using the Mann-Whitney-U or 2way-ANOVA with Sidak’s multiple comparison test (ns = not significant, * = p<0.05; ** = p<0.01).
(TIF)

S4 Fig IL-18 enhances the migratory capacity of NK cells. (a) Flow cytometric analysis of isolated cecal LP cells from 1:1 *Il18r1* ^-/-^—CD45.2: WT-CD45.1 mixed bone marrow chimeric mice, Sm-pretreated and infected orally with 5x10^7^ CFU *S*.Tm for 12h. NK1.1^+^ cell frequencies in blood from 1:1 mixed bone marrow chimeras; NK1.1^+^ cells are depicted as percentage of CD45.1^+^ or CD45.2^+^ blood cells. (b) Assessment of *in vivo* cell proliferation via EdU incorporation and flow cytometric analysis of isolated LP cells from C57BL/6 mice, either uninfected or infected orally with 5x10^7^CFU *S*.Tm for 12h (n = 5–6 per group). Quantification of EdU incorporation in CD3^-^ NK1.1^-^ CD11b^+^ cells. (c) Splenic NK cells were isolated by MACS sorting using the NK cell isolation kit II, NK cell purity was controlled by flow cytometry; shown are representative contour plots. (d) 2D Transwell migration assay of splenic NK cells from C57BL/6 WT mice (n = 3–4 per group) or (e) *Il18r1* ^-/-^ mice (n = 2–3 per group) and *Cxcr3* ^-/-^ mice (n = 2 per group). Splenic NK cells were isolated by MACS and stimulated for 3h in presence or absence of 100ng/mL rIL-18. Migration was performed towards indicated concentrations of CXCL9. Data represent the mean ± SD and statistical analysis was performed using 2way-ANOVA with Sidak’s multiple comparison test (ns = not significant, * = p<0.05; ** = p<0.01; *** = p<0.001).
(TIF)

S5 Fig Mucosal NK cells produce IFNγ but not TNF and GM-CSF during early *S*.Tm infection. (a and b) C57BL/6 mice were Sm-pretreated and infected for 12h with 5x10^7^ CFU *S*.Tm. (a) GM-CSF and (b) TNF protein level were measured in whole cecum tissue lysates by CBA; dashed lines indicate detection limit. (c) *Il18* ^-/-^ mice and littermates were Sm-pretreated and infected for 12h with 5x10^7^ CFU *S*.Tm. TNF protein level were measured in whole cecum tissue lysates by CBA; dashed line indicates detection limit. (d) C57BL/6 mice were Sm-pretreated and infected for 12h with 5x10^7^ CFU *S*.Tm. Flow cytometric analysis of TNF-producing cells, single CD45^+^ CD3^-^ CD19^-^ leukocytes were either gated on NK1.1^+^ cells (left) or NK1.1^-^ CD11b^+^ cells (right) and TNF production was assessed. Shown are representative dot plots of three independent experiments. (e) *Il18* ^-/-^ mice and littermate controls were Sm-pretreated and infected orally with 5x10^7^ CFU *S*.Tm for 18h. MFI of IFNγ signal in IFNγ-producing cells was determined by flow cytometric analysis of isolated cecal LP cells (n = 6 per group) (f) *Il18* ^-/-^ mice and littermates were infected for 18h with 5x10^7^ CFU *S*.Tm and treated with PBS or rIL-18 (120μg/kg, i.p.). Flow cytometric analysis of IFNγ-expressing cells, single lymphocytes were gated on CD45 and IFNg. (g) C57BL/6 mice were infected for 18h with 5x10^7^ CFU *S*.Tm and isolated cecal LP cells of four mice were pooled for staining and isotype control staining, data are shown from one out of two independent experiments. CD45^+^ NK1.1^+^ CD3^-^ IFNγ^+^ cells were characterized according to their surface expression of CD11b, Thy1, NKp46 and Eomes. (h) *Ifng* ^-/-^, *Ifngr1* ^-/-^ and littermate controls were Sm-pretreated, infected orally with 5x10^7^ CFU *S*.Tm for 12h and parameters of cecal pathology were assessed (grey bar- submucosal edema; dotted bar- goblet cells; white bar- PMN infiltrates; striped bar- tissue integrity). Data represent the mean ± SD and statistical analyses were performed using the Mann-Whitney-U or 2way-ANOVA with Sidak’s multiple comparison test (ns = not significant, * = p<0.05; ** = p<0.01).
(TIF)

S6 Fig Perforin deficiency affects cecal pathology but not cytokine production in the infected cecal mucosa. In an attempt to verify that NK cells indeed need a functional perforin response to elicit *S*.Tm induced mucosal pathology, we employed an immunodeficient mouse model, i.e. Rag2^-/-^γc^-/-^ mice that naturally lack T- and B cells as well as all innate lymphocyte subsets including NK cells. In mice with a similar defect (SCID/γc^-/-^) *S*.Tm is not able to induce proper inflammation [113]. We hypothesized that NK-cell transfer might rescue (at least partially) this defect in the host’s inflammatory response. This was tested experimentally: (a -c) *Prf1* ^-/-^ mice and littermates were Sm-pretreated and orally infected for 12h with 5x10^7^ CFU *S*.Tm. (a) *S*.Tm loads in cecum luminal content and (b) parameters of cecal pathology (grey bar- submucosal edema; dotted bar- goblet cells; white bar- PMN infiltrates; striped bar- tissue integrity) were assessed. (c) *Cxcl2*, *Ifng*, *Tnf*, *Il17a* and *S100a9* mRNA levels in whole cecum tissue were measured by RT-qPCR. Results are presented relative to the expression of *Actb*. (d -f) *Rag2* ^*-/-*^ *γc* ^*-/-*^ mice were transferred with either 5x10^5^ WT or 5x10^5^ *Prf* ^*-/-*^ isolated NK cells. 6 days later, they were infected with 5x10^7^ CFU *S*.Tm for 12h. (d) *S*.Tm loads in cecum luminal content, (e) histopathological score and (f) parameters of cecal pathology (grey bar- submucosal edema; dotted bar- goblet cells; white bar- PMN infiltrates; striped bar- tissue integrity). (g-i) C57BL/6 mice were Sm-pretreated, infected for 12h with 5x10^7^ CFU *S*.Tm and treated with 2.5mg Suramin at 6h p.i.. (g) *S*.Tm counts in cecum luminal content, (h) pathological score and (i) parameters of cecal pathology (grey bar- submucosal edema; dotted bar- goblet cells; white bar- PMN infiltrates; striped bar- tissue integrity). Statistical analysis was performed using the Mann-Whitney-U test (ns = not significant, * = p<0.05). Interpretation: 5 out of 8 *Rag2* ^-/-^ *γc* ^-/-^ mice transferred with WT NK cells showed borderline-to moderate mucosal inflammation. In contrast, no detectable inflammation was seen in any of the four mice transferred with *Prf1* ^-/-^ NK cells (S6e and S6f Fig). These data would support a functional importance of NK cell perforin expression in the induction of cecum pathology during early *S*.Tm infection. However, it is important to note that formally, we cannot exclude that the mucosal responses of *Rag2* ^-/-^ *γc* ^-/-^ mice are dominated by other pathways than those of WT animals. This may complicate the interpretation of the NK-cell transfer experiments with respect to the pro-inflammatory mechanisms that ramp up gut inflammation in WT mice.
(TIF)

S7 Fig Mucosal CD8 T cell response is not altered in *Il18* ^*-/-*^ animals during early *S*.Tm infection. (a–c) *Il18* ^*-/-*^ mice and littermates were Sm-pretreated, infected for 12h with 5x10^7^ CFU *S*.Tm and CD8 T cell counts were determined from isolated cecal LP. (a) gating strategy, (b) representative histograms of CD8α surface expression and (c) CD8 T cell numbers. (d-g) C57BL/6 mice were injected with anti-CD8 antibody (200μg/mouse; i.p.) 3 and 1 days prior to infection, animals were pretreated with Sm and infected orally with 5x10^7^ CFU *S*.Tm for 12h. (d) *S*.Tm loads in cecum luminal content, (e) representative histograms of CD8 T cell depletion efficiency in cecum and blood, (f) pathological score and (g) parameters of cecal pathology (grey bar- submucosal edema; dotted bar- goblet cells; white bar- PMN infiltrates; striped bar- tissue integrity). Data represent the mean ± SD and statistical analysis was performed using the Mann-Whitney-U test (ns = not significant).
(TIF)

S8 Fig Mucosal IL-18 is produced from both, the epithelial cell and the LP cell compartment. (a and b) IL-18-deficient mice, littermate controls and bone marrow chimeras (WT->IL18: WT BM into IL-18 deficient mice; IL18->WT: *Il18* ^-/-^ BM into WT mice) were Sm-pretreated and infected orally with 5x10^7^ CFU *S*.Tm. (a) Histopathological score of bone marrow chimeras and (b) mature IL-18 levels measured in full cecum tissue lysate. Data represent the mean ± SD and statistical analyses were performed using the Mann-Whitney-U or 1way-ANOVA with Sidak’s multiple comparison test (ns = not significant, * = p<0.05).
(TIF)

S1 Table List of genes significantly downregulated in *Il18* ^*-/-*^ compared to WT animals. (XLSX)

S1 References Supporting References. (DOCX)
