# Supplementary figures and images for: An NK Cell Perforin Response Elicited via IL-18 Controls Mucosal Inflammation Kinetics during Salmonella Gut Infection
Source: PLoS Pathog. 2016 Jun 24;12(6):e1005723. doi: 10.1371/journal.ppat.1005723 (PMC4920399; doi:10.1371/journal.ppat.1005723)

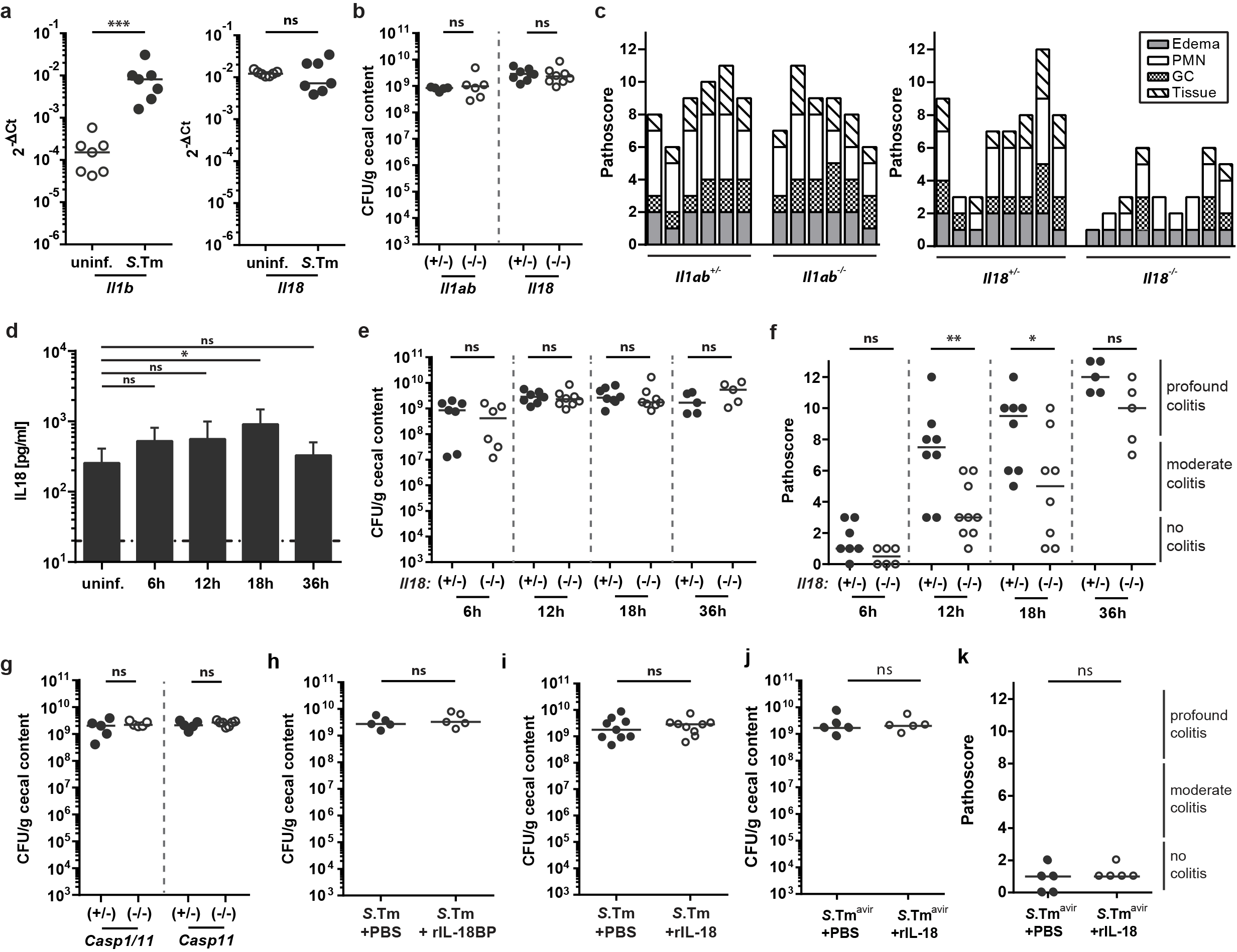

Supplement: S1 Fig — (a) C57BL/6 WT mice were Sm-pretreated and either uninfected or infected orally with 5x10^7^ CFU S.Tm for 12h (n = 7 per group). Il1b and Il18 mRNA levels in whole cecum tissue were measured by RT-qPCR. Results are presented relative to the expression of Actb. (b and c) Il1ab -/- and Il18 -/- mice and littermate controls were Sm-pretreated and infected orally with 5x10^7^ CFU S.Tm for 12h (n = 6–9 per group). (b) S.Tm loads in cecum luminal contents and (c) parameters of cecal pathology (grey bar- submucosal edema; dotted bar- goblet cells; white bar- PMN infiltrates; striped bar- tissue integrity). (d-f) Il18 -/- mice and littermates were Sm-pretreated and infected orally with 5x10^7^ CFU S.Tm for the indicated time points (n = 5–9 per group). (d) IL-18 protein levels in whole cecum tissue lysates from Il18 littermates (n = 5–6 per group); dashed line indicates detection limit. (e) S.Tm load in cecum luminal content and (f) pathological score. Note that 12h data are replotted from panel 1b and S1b. (g) Casp1/11 -/- and Casp11 -/- mice and littermate controls were Sm-pretreated, infected orally with 5x10^7^ CFU S.Tm for 12h (n = 5–7 per group) and S.Tm load in cecum luminal content was assessed. (h) C57BL/6 WT mice were Sm-pretreated, injected intraperitoneally with rIL-18BP or PBS, infected orally with 5x10^7^ CFU S.Tm for 12h (n = 5 per group) and S.Tm load in cecum luminal content was assessed. (i) C57BL/6 WT mice were Sm-pretreated, injected intraperitoneally with rIL18 or PBS, infected orally with 5x10^7^ CFU S.Tm for 8h (n = 9 per group) and cecum luminal content was assessed. (j and k) C57BL/6 WT mice were Sm-pretreated, injected intraperitoneally with rIL18 or PBS and infected orally with 1x10^10^ CFU S.Tmavir for 12h (n = 5 per group). (j) S.Tmavir load in luminal content, (k) pathological score. Statistical analyses were performed using the Mann-Whitney-U test or 1way-ANOVA with Sidak's multiple comparison test (ns = not significant, * = p<0.05; ** = p<0.01; *** = p<0.0 [file ppat.1005723.s001.tif]

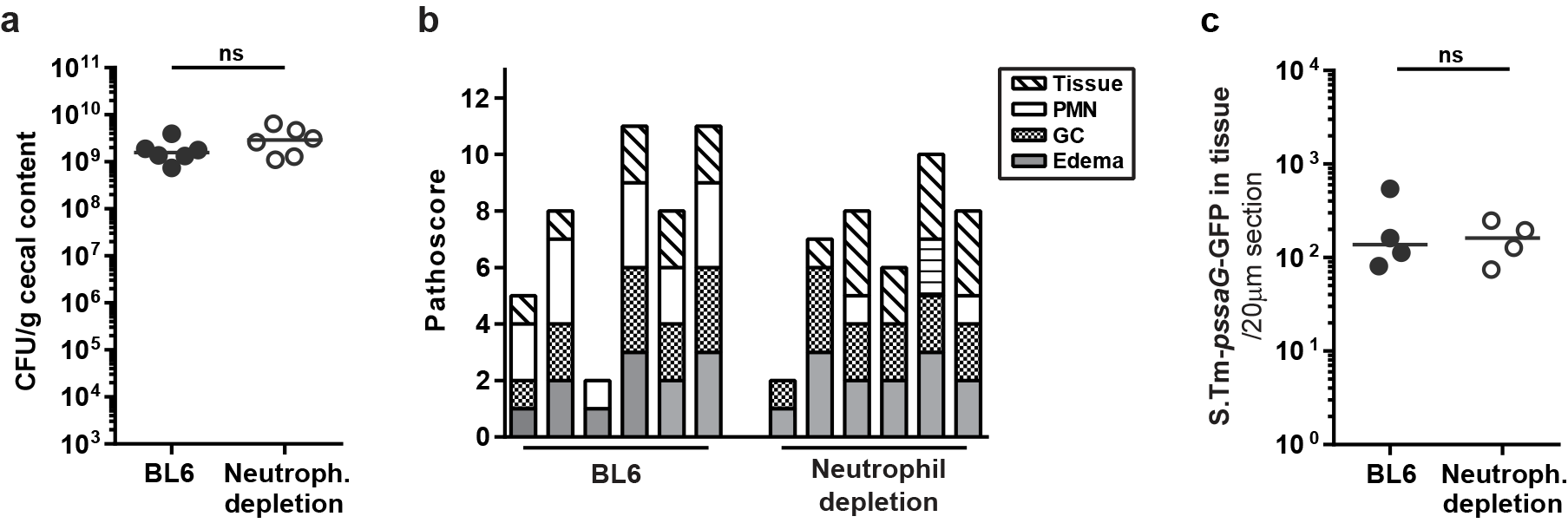

Supplement: S2 Fig — (a and b) C57BL/6 WT mice were injected intraperitoneally with anti-G-CSF (0.4mg/kg; two consecutive days) and anti-Ly-6G (6mg/kg; two days prior to infection) or PBS. Mice were Sm-pretreated and infected orally with 5x10^7^ CFU S.Tm for 12h (n = 6 per group). (a) S.Tm loads in cecum luminal content and (b) parameters of cecal pathology (grey bar- submucosal edema; dotted bar- goblet cells; white bar- PMN infiltrates; striped bar- tissue integrity). (c) C57BL/6 WT mice were injected intraperitoneally with anti-G-CSF (0.4mg/kg; two consecutive days) and anti-Ly-6G (6mg/kg; two days prior to infection) or PBS. Mice were Sm-pretreated, infected orally with 5x10^7^ CFU S.Tm-pssaG-GFPmut2 for 12h (n = 4 per group) and S.Tm cecum tissue counts were determined per 20μm cross-section. Statistical analysis was performed using the Mann-Whitney-U test (ns = not significant). (TIF) [file ppat.1005723.s002.tif]

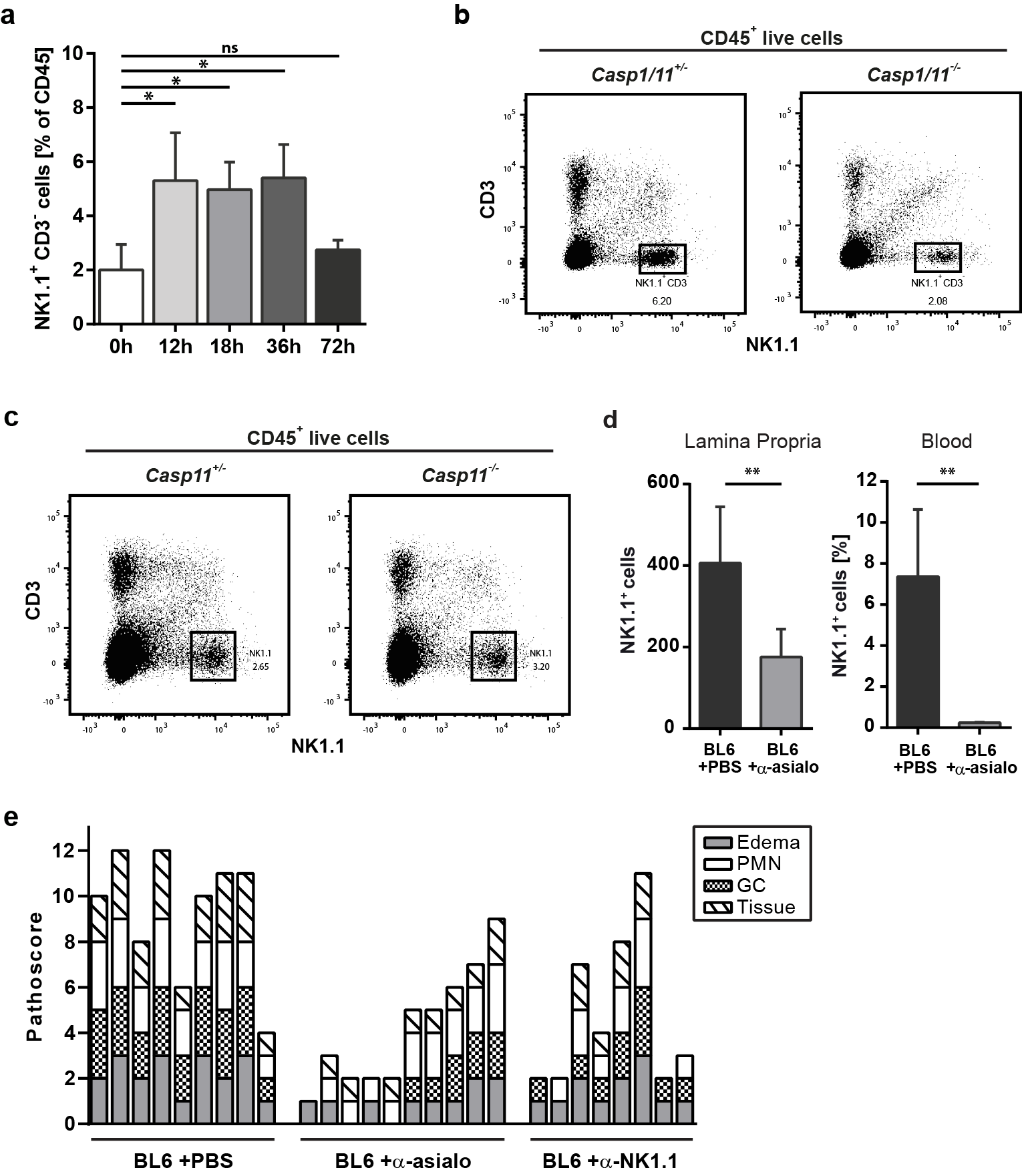

Supplement: S3 Fig — (a) Flow cytometric analysis of isolated cecal LP cells from Sm-pretreated C57BL/6 WT mice, either uninfected or infected with 5x10^7^ CFU S.Tm for the indicated time points (n = 4–5 per group). Single live cells were gated on CD45+ lymphocytes and NK1.1+ CD3- cells were quantified. (b and c) Flow cytometric analysis of isolated cecal LP cells from Sm-pretreated (b) Casp1/11-/- or (c) Casp11-/- mice. Single live CD45+ lymphocytes were gated on CD45+ NK1.1+ CD3- cells, shown are representative dot plots. (d) C57BL/6 WT mice were injected intraperitoneally with anti-asialo GM1 antiserum or PBS and mice were infected orally with 5x10^7^ CFU S.Tm for 12h (n = 10 per group). Depletion efficiency of NK1.1+ cells in (left) cecum and (right) blood. (e) C57BL/6 WT mice were injected intraperitoneally with anti-asialo GM1 antiserum (50μL antiserum/mouse; three consecutive days), anti-NK1.1 (10mg/kg; 2 consecutive days) or PBS and were infected orally with 5x10^7^ CFU S.Tm for 12h (n = 8–10 per group). Parameters of cecal pathology were assessed (grey bar- submucosal edema; dotted bar- goblet cells; white bar- PMN infiltrates; striped bar- tissue integrity). Data represent the mean ± SD and statistical analyses were performed using the Mann-Whitney-U or 2way-ANOVA with Sidak’s multiple comparison test (ns = not significant, * = p<0.05; ** = p<0.01). (TIF) [file ppat.1005723.s003.tif]

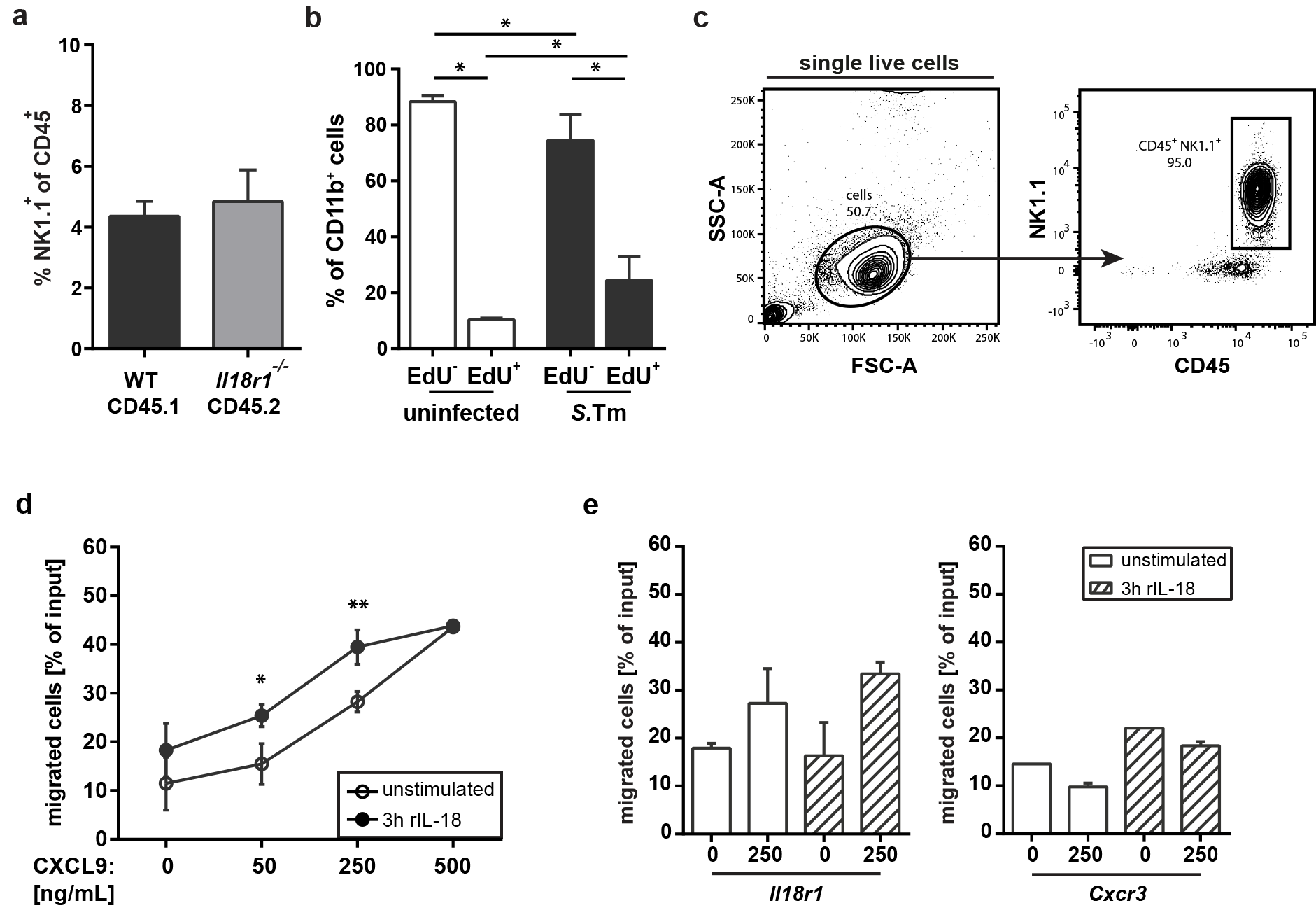

Supplement: S4 Fig — (a) Flow cytometric analysis of isolated cecal LP cells from 1:1 Il18r1 -/-—CD45.2: WT-CD45.1 mixed bone marrow chimeric mice, Sm-pretreated and infected orally with 5x10^7^ CFU S.Tm for 12h. NK1.1+ cell frequencies in blood from 1:1 mixed bone marrow chimeras; NK1.1+ cells are depicted as percentage of CD45.1+ or CD45.2+ blood cells. (b) Assessment of in vivo cell proliferation via EdU incorporation and flow cytometric analysis of isolated LP cells from C57BL/6 mice, either uninfected or infected orally with 5x10^7^CFU S.Tm for 12h (n = 5–6 per group). Quantification of EdU incorporation in CD3- NK1.1- CD11b+ cells. (c) Splenic NK cells were isolated by MACS sorting using the NK cell isolation kit II, NK cell purity was controlled by flow cytometry; shown are representative contour plots. (d) 2D Transwell migration assay of splenic NK cells from C57BL/6 WT mice (n = 3–4 per group) or (e) Il18r1 -/- mice (n = 2–3 per group) and Cxcr3 -/- mice (n = 2 per group). Splenic NK cells were isolated by MACS and stimulated for 3h in presence or absence of 100ng/mL rIL-18. Migration was performed towards indicated concentrations of CXCL9. Data represent the mean ± SD and statistical analysis was performed using 2way-ANOVA with Sidak’s multiple comparison test (ns = not significant, * = p<0.05; ** = p<0.01; *** = p<0.001). (TIF) [file ppat.1005723.s004.tif]

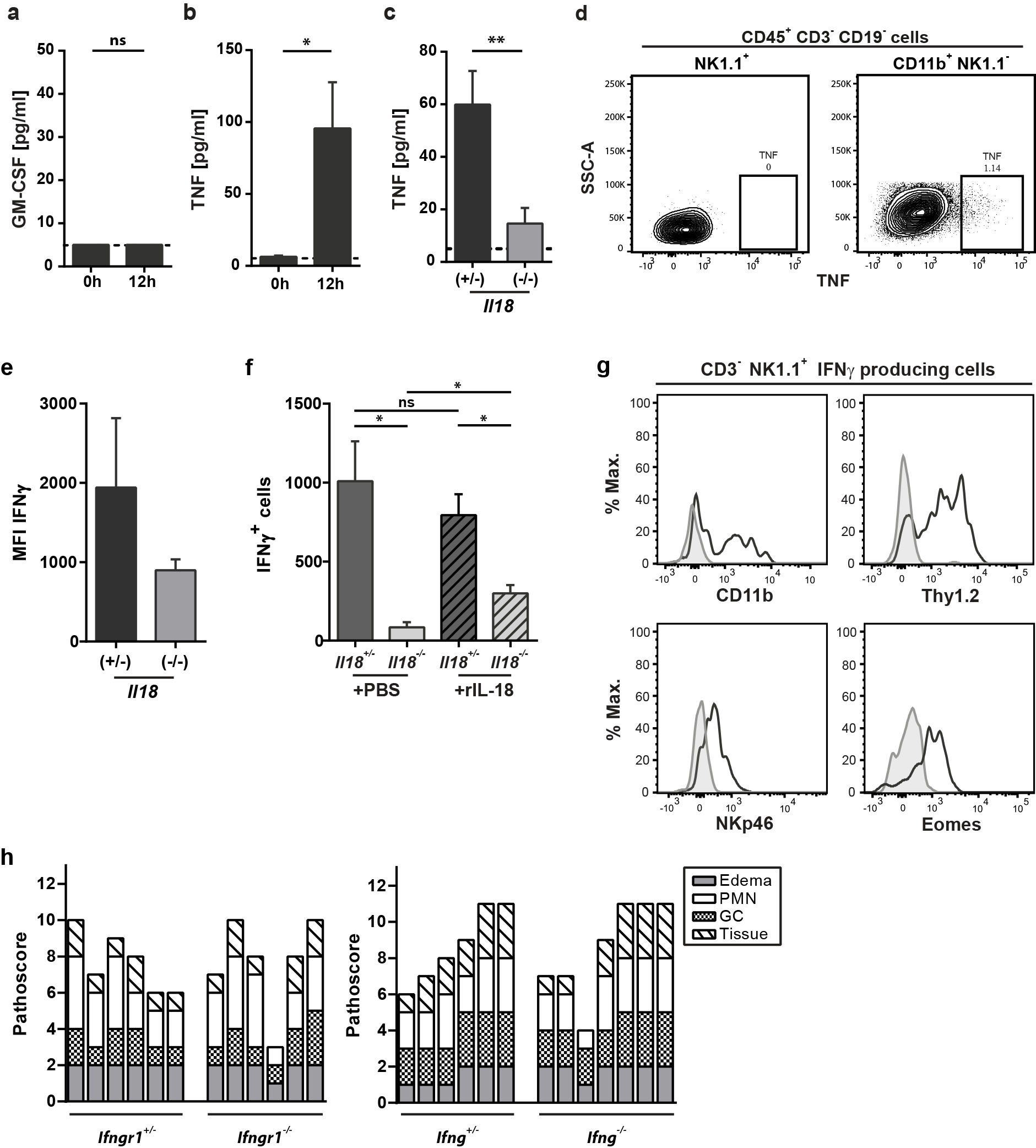

Supplement: S5 Fig — (a and b) C57BL/6 mice were Sm-pretreated and infected for 12h with 5x10^7^ CFU S.Tm. (a) GM-CSF and (b) TNF protein level were measured in whole cecum tissue lysates by CBA; dashed lines indicate detection limit. (c) Il18 -/- mice and littermates were Sm-pretreated and infected for 12h with 5x10^7^ CFU S.Tm. TNF protein level were measured in whole cecum tissue lysates by CBA; dashed line indicates detection limit. (d) C57BL/6 mice were Sm-pretreated and infected for 12h with 5x10^7^ CFU S.Tm. Flow cytometric analysis of TNF-producing cells, single CD45+ CD3- CD19- leukocytes were either gated on NK1.1+ cells (left) or NK1.1- CD11b+ cells (right) and TNF production was assessed. Shown are representative dot plots of three independent experiments. (e) Il18 -/- mice and littermate controls were Sm-pretreated and infected orally with 5x10^7^ CFU S.Tm for 18h. MFI of IFNγ signal in IFNγ-producing cells was determined by flow cytometric analysis of isolated cecal LP cells (n = 6 per group) (f) Il18 -/- mice and littermates were infected for 18h with 5x10^7^ CFU S.Tm and treated with PBS or rIL-18 (120μg/kg, i.p.). Flow cytometric analysis of IFNγ-expressing cells, single lymphocytes were gated on CD45 and IFNg. (g) C57BL/6 mice were infected for 18h with 5x10^7^ CFU S.Tm and isolated cecal LP cells of four mice were pooled for staining and isotype control staining, data are shown from one out of two independent experiments. CD45+ NK1.1+ CD3- IFNγ+ cells were characterized according to their surface expression of CD11b, Thy1, NKp46 and Eomes. (h) Ifng -/-, Ifngr1 -/- and littermate controls were Sm-pretreated, infected orally with 5x10^7^ CFU S.Tm for 12h and parameters of cecal pathology were assessed (grey bar- submucosal edema; dotted bar- goblet cells; white bar- PMN infiltrates; striped bar- tissue integrity). Data represent the mean ± SD and statistical analyses were performed using the Mann-Whitney-U or 2way-ANOVA with Sidak’s multiple comparison test (ns = not significant, * = [file ppat.1005723.s005.tif]

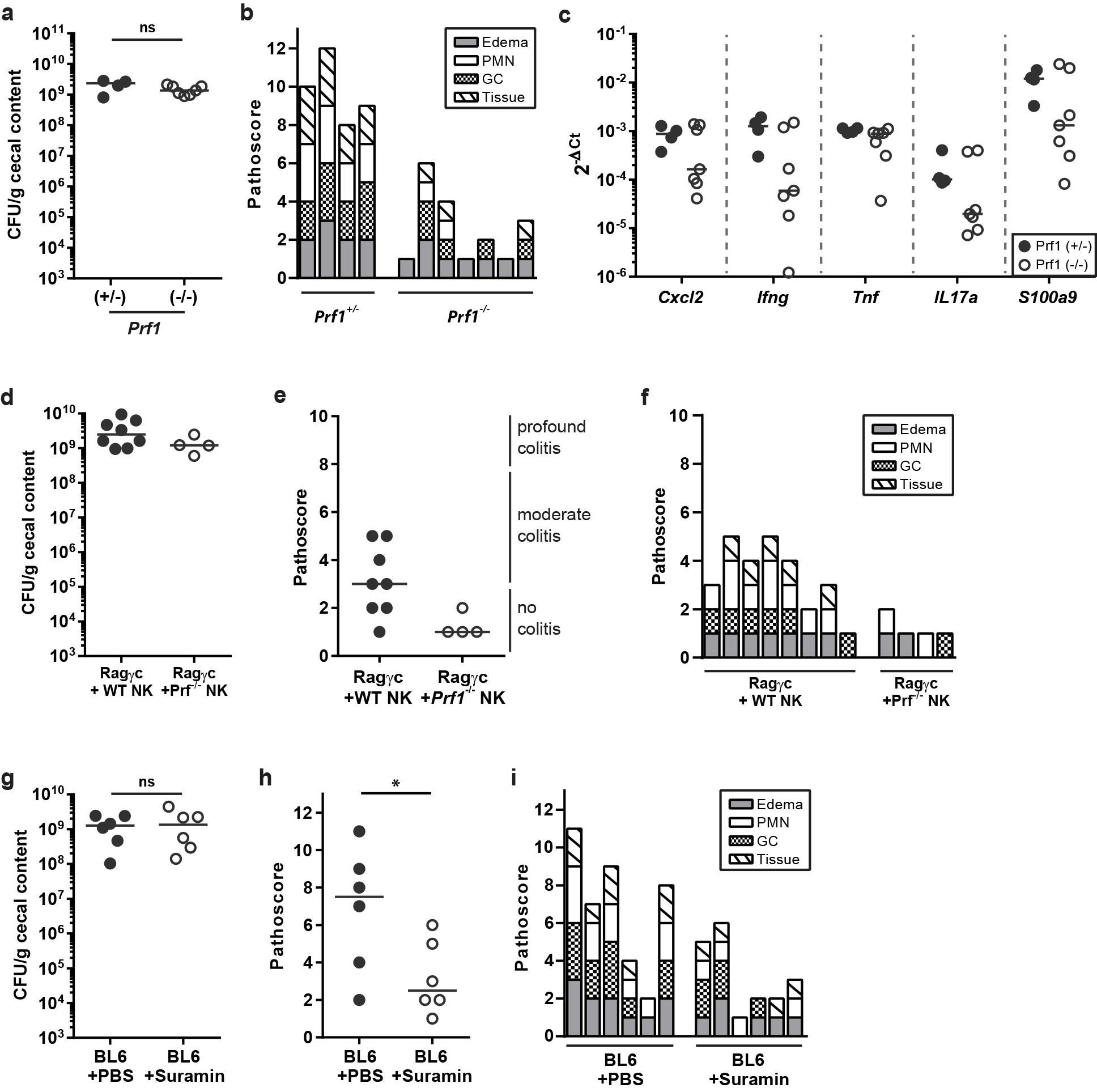

Supplement: S6 Fig — In an attempt to verify that NK cells indeed need a functional perforin response to elicit S.Tm induced mucosal pathology, we employed an immunodeficient mouse model, i.e. Rag2-/-γc-/- mice that naturally lack T- and B cells as well as all innate lymphocyte subsets including NK cells. In mice with a similar defect (SCID/γc-/-) S.Tm is not able to induce proper inflammation [113]. We hypothesized that NK-cell transfer might rescue (at least partially) this defect in the host’s inflammatory response. This was tested experimentally: (a -c) Prf1 -/- mice and littermates were Sm-pretreated and orally infected for 12h with 5x10^7^ CFU S.Tm. (a) S.Tm loads in cecum luminal content and (b) parameters of cecal pathology (grey bar- submucosal edema; dotted bar- goblet cells; white bar- PMN infiltrates; striped bar- tissue integrity) were assessed. (c) Cxcl2, Ifng, Tnf, Il17a and S100a9 mRNA levels in whole cecum tissue were measured by RT-qPCR. Results are presented relative to the expression of Actb. (d -f) Rag2 -/- γc -/- mice were transferred with either 5x10^5^ WT or 5x10^5^ Prf -/- isolated NK cells. 6 days later, they were infected with 5x10^7^ CFU S.Tm for 12h. (d) S.Tm loads in cecum luminal content, (e) histopathological score and (f) parameters of cecal pathology (grey bar- submucosal edema; dotted bar- goblet cells; white bar- PMN infiltrates; striped bar- tissue integrity). (g-i) C57BL/6 mice were Sm-pretreated, infected for 12h with 5x10^7^ CFU S.Tm and treated with 2.5mg Suramin at 6h p.i.. (g) S.Tm counts in cecum luminal content, (h) pathological score and (i) parameters of cecal pathology (grey bar- submucosal edema; dotted bar- goblet cells; white bar- PMN infiltrates; striped bar- tissue integrity). Statistical analysis was performed using the Mann-Whitney-U test (ns = not significant, * = p<0.05). Interpretation: 5 out of 8 Rag2 -/- γc -/- mice transferred with WT NK cells showed borderline-to moderate mucosal inflammation. In contrast, no detectable inflammation wa [file ppat.1005723.s006.tif]

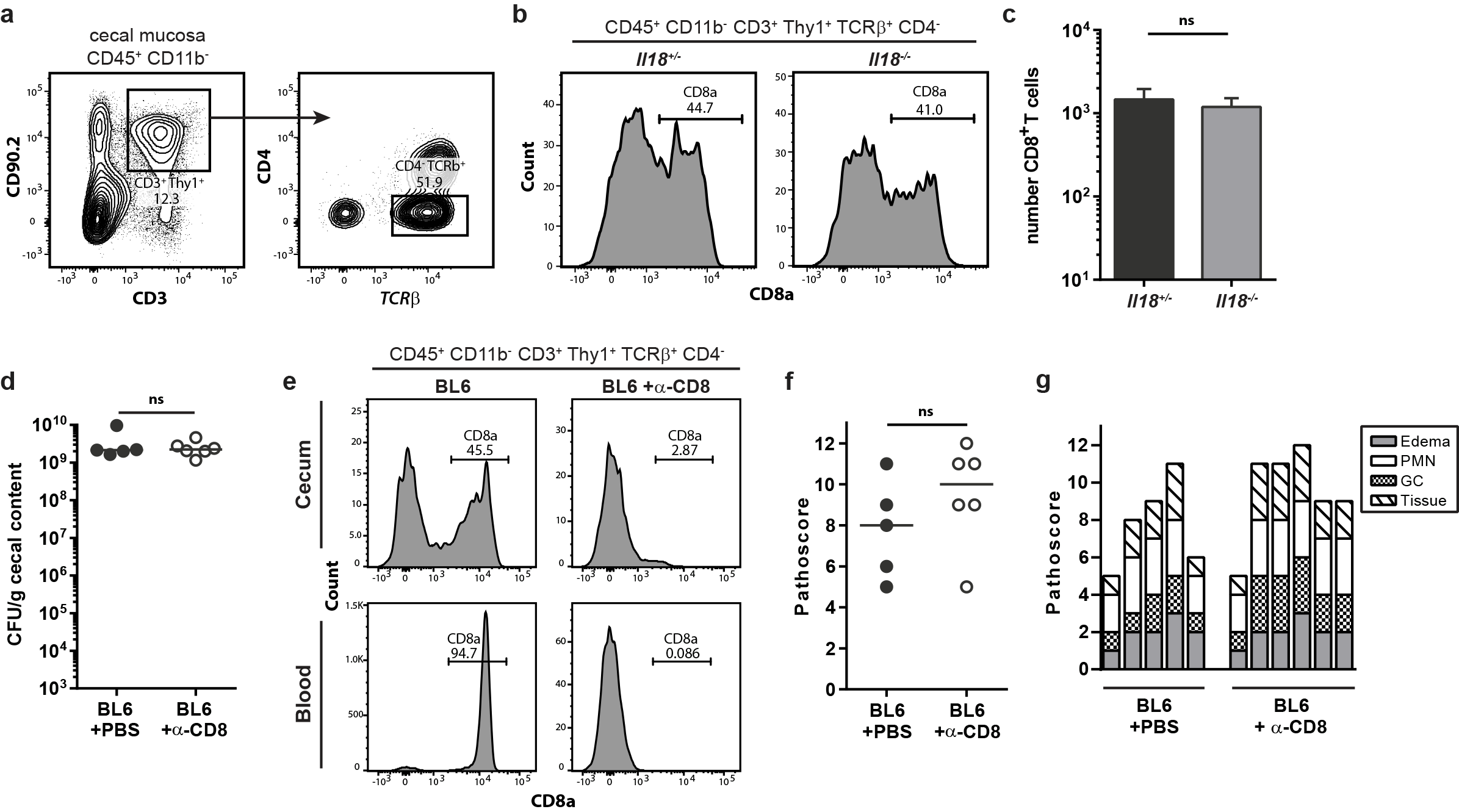

Supplement: S7 Fig — (a–c) Il18 -/- mice and littermates were Sm-pretreated, infected for 12h with 5x10^7^ CFU S.Tm and CD8 T cell counts were determined from isolated cecal LP. (a) gating strategy, (b) representative histograms of CD8α surface expression and (c) CD8 T cell numbers. (d-g) C57BL/6 mice were injected with anti-CD8 antibody (200μg/mouse; i.p.) 3 and 1 days prior to infection, animals were pretreated with Sm and infected orally with 5x10^7^ CFU S.Tm for 12h. (d) S.Tm loads in cecum luminal content, (e) representative histograms of CD8 T cell depletion efficiency in cecum and blood, (f) pathological score and (g) parameters of cecal pathology (grey bar- submucosal edema; dotted bar- goblet cells; white bar- PMN infiltrates; striped bar- tissue integrity). Data represent the mean ± SD and statistical analysis was performed using the Mann-Whitney-U test (ns = not significant). (TIF) [file ppat.1005723.s007.tif]

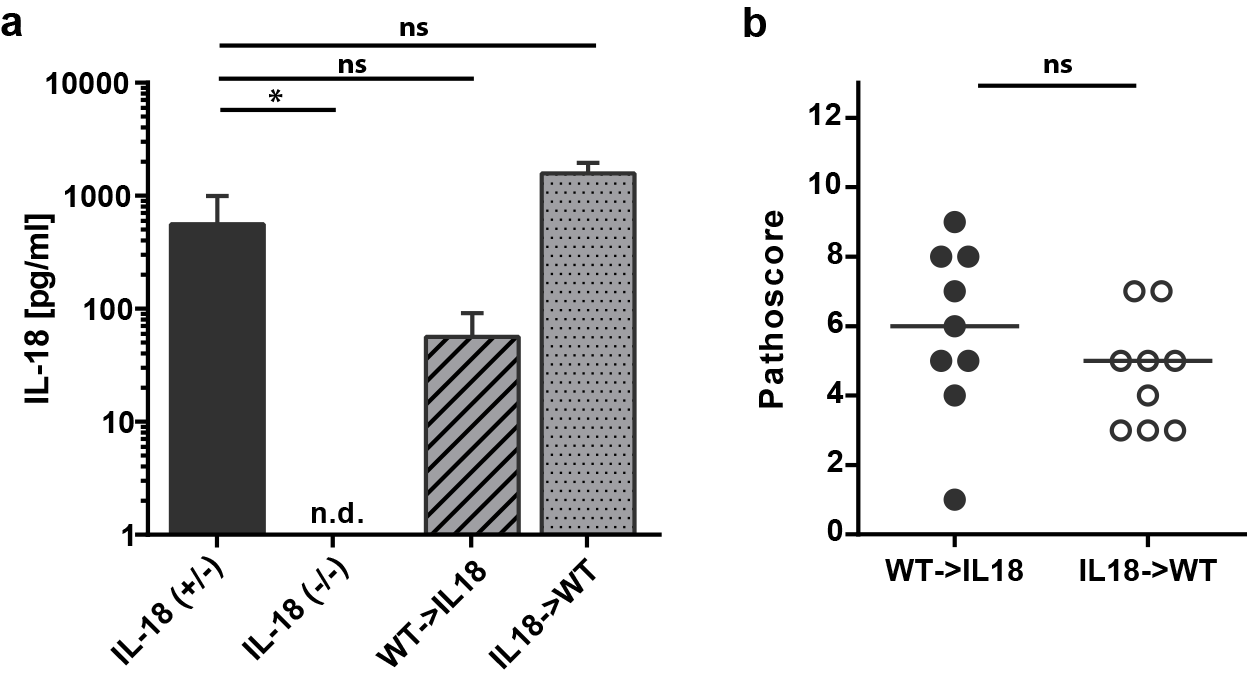

Supplement: S8 Fig — (a and b) IL-18-deficient mice, littermate controls and bone marrow chimeras (WT->IL18: WT BM into IL-18 deficient mice; IL18->WT: Il18 -/- BM into WT mice) were Sm-pretreated and infected orally with 5x10^7^ CFU S.Tm. (a) Histopathological score of bone marrow chimeras and (b) mature IL-18 levels measured in full cecum tissue lysate. Data represent the mean ± SD and statistical analyses were performed using the Mann-Whitney-U or 1way-ANOVA with Sidak’s multiple comparison test (ns = not significant, * = p<0.05). (TIF) [file ppat.1005723.s008.tif]
