# Supplementary material for: An NK Cell Perforin Response Elicited via IL-18 Controls Mucosal Inflammation Kinetics during Salmonella Gut Infection
Source: PLoS Pathog. 2016 Jun 24;12(6):e1005723. doi: 10.1371/journal.ppat.1005723 (PMC4920399; doi:10.1371/journal.ppat.1005723)
Supplement: S1 References — (DOCX) [file ppat.1005723.s010.docx]

**S1 References**

113. Harrington, L., Srikanth, C.V., Antony, R., Shi, H.N., Cherayil, B.J. A role for natural killer cells in intestinal inflammation caused by infection with Salmonella enterica serovar Typhimurium. *FEMS Immunol Med Microbiol* **51**,372-380(2007).
